# Polycomb repressive complex 2 facilitates the transition from heterotrophy to photoautotrophy during seedling emergence

**DOI:** 10.1093/plcell/koaf148

**Published:** 2025-06-14

**Authors:** Naseem Samo, María Guadalupe Trejo-Arellano, Lenka Gahurová, Alexander Erban, Alina Ebert, Quentin Rivière, Jiří Kubásek, Fatemeh Aflaki, Helena Hönig Mondeková, Armin Schlereth, Annick Dubois, Mingxi Zhou, Ondřej Novák, Jiří Šantrůček, Daniel Bouyer, François Roudier, Joachim Kopka, Iva Mozgová

**Affiliations:** Biology Centre of the Czech Academy of Sciences, Institute of Plant Molecular Biology, Branišovská 31, České Budějovice 37005, Czech Republic; University of South Bohemia in České Budějovice, Faculty of Science, Branišovská 31, České Budějovice 37005, Czech Republic; Biology Centre of the Czech Academy of Sciences, Institute of Plant Molecular Biology, Branišovská 31, České Budějovice 37005, Czech Republic; University of South Bohemia in České Budějovice, Faculty of Science, Branišovská 31, České Budějovice 37005, Czech Republic; Applied Metabolome Analysis Infrastructure Group, Max Planck Institute of Molecular Plant Physiology, Am Mühlenberg 1, Potsdam 14476, Germany; Applied Metabolome Analysis Infrastructure Group, Max Planck Institute of Molecular Plant Physiology, Am Mühlenberg 1, Potsdam 14476, Germany; Biology Centre of the Czech Academy of Sciences, Institute of Plant Molecular Biology, Branišovská 31, České Budějovice 37005, Czech Republic; University of South Bohemia in České Budějovice, Faculty of Science, Branišovská 31, České Budějovice 37005, Czech Republic; Biology Centre of the Czech Academy of Sciences, Institute of Plant Molecular Biology, Branišovská 31, České Budějovice 37005, Czech Republic; Biology Centre of the Czech Academy of Sciences, Institute of Plant Molecular Biology, Branišovská 31, České Budějovice 37005, Czech Republic; University of South Bohemia in České Budějovice, Faculty of Science, Branišovská 31, České Budějovice 37005, Czech Republic; Applied Metabolome Analysis Infrastructure Group, Max Planck Institute of Molecular Plant Physiology, Am Mühlenberg 1, Potsdam 14476, Germany; Laboratoire Reproduction et Développement des Plantes, Université de Lyon, ENS de Lyon, CNRS, INRAE, INRIA, 69342 Lyon, France; Biology Centre of the Czech Academy of Sciences, Institute of Plant Molecular Biology, Branišovská 31, České Budějovice 37005, Czech Republic; Laboratory of Growth Regulators, Faculty of Science of Palacký University and Institute of Experimental Botany of the Czech Academy of Sciences, Šlechtitelů 27, Olomouc 78371, Czech Republic; University of South Bohemia in České Budějovice, Faculty of Science, Branišovská 31, České Budějovice 37005, Czech Republic; Laboratoire Reproduction et Développement des Plantes, Université de Lyon, ENS de Lyon, CNRS, INRAE, INRIA, 69342 Lyon, France; Laboratoire Reproduction et Développement des Plantes, Université de Lyon, ENS de Lyon, CNRS, INRAE, INRIA, 69342 Lyon, France; Applied Metabolome Analysis Infrastructure Group, Max Planck Institute of Molecular Plant Physiology, Am Mühlenberg 1, Potsdam 14476, Germany; Biology Centre of the Czech Academy of Sciences, Institute of Plant Molecular Biology, Branišovská 31, České Budějovice 37005, Czech Republic; University of South Bohemia in České Budějovice, Faculty of Science, Branišovská 31, České Budějovice 37005, Czech Republic

## Abstract

The seed-to-seedling transition represents a key developmental and metabolic switch in plants. Catabolism of seed storage reserves fuels germination and early seedling emergence until photosynthesis is established. The seed-to-seedling developmental transition is controlled by Polycomb repressive complex 2 (PRC2). However, the coordination of PRC2 activity and its contribution to transcriptional reprogramming during seedling establishment remain unknown. By analyzing H3K27me3 re-distribution and changes in gene transcription in the shoot and root tissues of heterotrophic and photoautotrophic Arabidopsis (*Arabidopsis thaliana*) seedlings, we reveal 2 phases of PRC2-mediated gene repression. The first phase is independent of light and photosynthesis and results in the irreversible repression of the embryo maturation program, marked by heterotrophy and reserve storage molecule biosynthesis. The second phase is associated with the repression of metabolic pathways related to germination and early seedling emergence, and H3K27me3 deposition in this phase is sensitive to photosynthesis inhibition. We show that preventing the transcription of the PRC2-repressed glyoxylate cycle gene *ISOCITRATE LYASE* promotes the vegetative phase transition in PRC2-depleted plants. Our findings underscore a key role of PRC2-mediated transcriptional repression in the coordinated metabolic and developmental switches that occur during seedling emergence and emphasize the close connection between metabolic and developmental identities.

## Teaser

Two phases of gene repression by Polycomb repressive complex 2 are associated with metabolic and developmental transition during seedling establishment in Arabidopsis.

## Introduction

Seedling establishment represents an important developmental and metabolic transition in plants. Seed germination and early seedling growth are fueled by the catabolism of seed storage reserves before the onset of photosynthesis (Graham 2008; Tan-Wilson and Wilson 2012), completing the transition from the heterotrophic to the photoautotrophic growth phase. In Arabidopsis (*Arabidopsis thaliana*), acyl lipids—triacylglycerols (TAGs) are the major seed storage molecules (Li-Beisson et al. 2013). TAGs are broken down by lipases into free fatty acids (FAs) and glycerol (Quettier and Eastmond 2009). FAs are then converted into acetyl-coenzyme A (Ac-CoA) via β-oxidation (Graham 2008). Ac-CoA enters the glyoxylate cycle and is converted into 4-carbon compounds by the activities of enzymes including isocitrate lyase (ICL) and malate synthase (Graham 2008 ). These compounds are then transported to the mitochondria, where they can either be processed and transported to the cytosol for gluconeogenesis or used as substrates for respiration.

Seed-stored reserves are used during shoot axis (hypocotyl) elongation following seed germination in the soil (i.e. in darkness), where root growth remains suppressed (skotomorphogenesis). Once the hypocotyl reaches light, its elongation ceases and the cotyledons become photosynthetically active (photomorphogenesis) (Josse and Halliday 2008; Arsovski et al. 2012). Light signaling and photosynthesis-derived sugars are required to activate the shoot and root apical meristems and to promote cell division and growth of postembryonic organs (Kircher and Schopfer 2012; Xiong et al. 2013; Pfeiffer et al. 2016). Seed germination and photomorphogenesis in Arabidopsis involve extensive reprogramming of gene expression (Silva et al. 2016; Narsai et al. 2017 ; Pan et al. 2023; Tremblay et al. 2024) associated with global and local reorganization of chromatin (Van Zanten et al. 2012; Bourbousse et al. 2020; Simon and Probst 2024) and changes in distribution of several histone modifications including H2Bub (Bourbousse et al. 2012), H3K9ac, H3K9me3, H3K27ac, and H3K27me3 (Charron et al. 2009; Pan et al. 2023). Polycomb repressive complexes (PRCs) PRC1 and PRC2, which catalyze H2AK121ub (H2Aub) and H3K27me3, respectively, are required for the seed-to-seedling transition (Aichinger et al. 2009 ; Baile et al. 2022; Bouyer et al. 2011; Bratzel et al. 2010 ; Chanvivattana et al. 2004; Chen et al. 2010; Yang et al. 2013). Deposition of H2Aub or H3K27me3 at the embryo transcription factor genes (*TFs*) *LEAFY COTYLEDON1* (*LEC1*), *LEC1-LIKE, ABSCISIC ACID INSENSITIVE 3* (*ABI3*), *FUSCA3* (*FUS3*), *LEC2* (or collectively “*LAFLs*”) (Bratzel et al. 2010 ; Chen et al. 2010; Yang et al. 2013; Jia et al. 2014; Lepiniec et al. 2018; Gazzarrini and Song 2024), *AGAMOUS-LIKE 15* (*AGL15*) (Chen et al. 2018), and dormancy regulator *DELAY OF GERMINATION1* (*DOG1*) (Molitor et al. 2014 ; Chen et al. 2020 ) leads to their stable repression, facilitating seed germination and seedling establishment. Accordingly, the absence of the PRC1 catalytic subunits *A. THALIANA* B-lymphoma Mo-MLV insertion region 1 (AtBMI1) or *A. THALIANA* REALLY INTERESTING NEW GENE 1A (AtRING1), the PRC2 catalytic subunits CURLY LEAF (CLF) and SWINGER (SWN), or the PRC2 WD40 subunit FERTILIZATION INDEPENDENT ENDOSPERM (FIE), results in delayed seed germination, failure to develop differentiated tissues, accumulation of TAGs and development of somatic embryos (Chanvivattana et al. 2004; Aichinger et al. 2009; Bratzel et al. 2010 ; Chen et al. 2010; Bouyer et al. 2011; Yang et al. 2013 ; Ikeuchi et al. 2015; Mozgová et al. 2017).

The recruitment of PRC2 to target loci in Arabidopsis is facilitated by distinct families of PRC2-interacting TFs that recognize promoter elements, or Polycomb response elements (PREs), within the target gene promoters (Xiao et al. 2017 ). Among them, the B3 domain TFs VIVIPAROUS-1/ABSCISIC ABI3-LIKE VAL1/VAL2, which bind the RY element (CATGCA/TGCATG) (Yuan et al. 2021), the telobox-binding TFs TELOMERE REPEAT BINDING PROTEIN1 (TRB1) (Zhou et al. 2018) and ARABIDOPSIS ZINC FINGER 1 (AZF1) (Xiao et al. 2017), and the GA_n_-motif-binding BASIC PENTACYSTEINE (BPC) TFs (Xiao et al. 2017) are responsible for genome-wide PRC2 targeting. *LEC2* PRE-like element is required for the deposition of H3K27me3 and *LEC2* repression in vegetative seedlings (Berger et al. 2011). VAL1 and VAL2 recruit components of the PRC2 to the promoter of *AGL15* (Chen et al. 2018) and to *DOG1* (Chen et al. 2020), facilitating their repression. The establishment of H3K27me3 at *LAFLs* is also dependent of VAL1/VAL2 and H2Aub (Yang et al. 2013). VAL1 interacts with the components of histone deacetylase complex (HDAC), PRC1 and also recruits PRC2, which suggests its leading role in establishing a repressed state of the underlying genes (Yang et al. 2013; Questa et al. 2016; Baile et al. 2021; Mikulski et al. 2022). The temporal dynamics of H3K27me3 deposition and its regulation during seedling emergence is largely unknown. During seed germination and early seedling emergence, H3K27me3 demethylase RELATIVE OF EARLY FLOWERING 6 (REF6) maintains a demethylated state at seedling establishment genes, promoting their expression and seed germination (Pan et al. 2023). Genes encoding PRC2 subunits are transcriptionally activated after seed imbibition (Mozgová et al. 2017; Pan et al. 2023), and changes in H3K27me3 distribution are initiated at 24 to 48 h (hrs) after seed imbibition (Pan et al. 2023). The nuclear activity of PRC2 is promoted by the kinase target of rapamycin (TOR) (Ye et al. 2022), an important integrator of metabolic and nutrient state that balances cell division and growth versus stress response (Ryabova et al. 2019; Wu et al. 2019). However, it remains unknown how the activity of PRC2 and reprogramming of H3K27me3 during seedling emergence alter gene expression to achieve a stable developmental and metabolic transition during seedling establishment.

We show that PRC2 is required to stably repress heterotrophic growth and initiate photoautotrophic (vegetative) growth and development. By profiling the transcriptome and H3K27me3 distribution in wild type (WT) and PRC2-catalytic double mutant seedlings *clf swn* (*cs*) at 2 metabolically distinct developmental timepoints, we identified 2 phases of H3K27me3 deposition that underlie a stable transition to vegetative growth. In the first phase, H3K27me3 deposition is independent of light and photosynthesis. It ensures the repression of genes that contribute to a sugar-inducible activation of developmental and metabolic pathways involved in embryo maturation. In the second phase, metabolic processes associated with seed germination/early seedling establishment and early light responses are repressed. By preventing overexpression of the PRC2-target *ICL* in *cs*, we show that PRC2-mediated transcriptional repression of metabolic genes during germination promotes the developmental transition from seed to seedling.

## Results

### Shoot and root tissues of seedlings before and after the onset of carbon assimilation show different transcriptome and H3K27me3 distribution profiles

**Figure 1.**
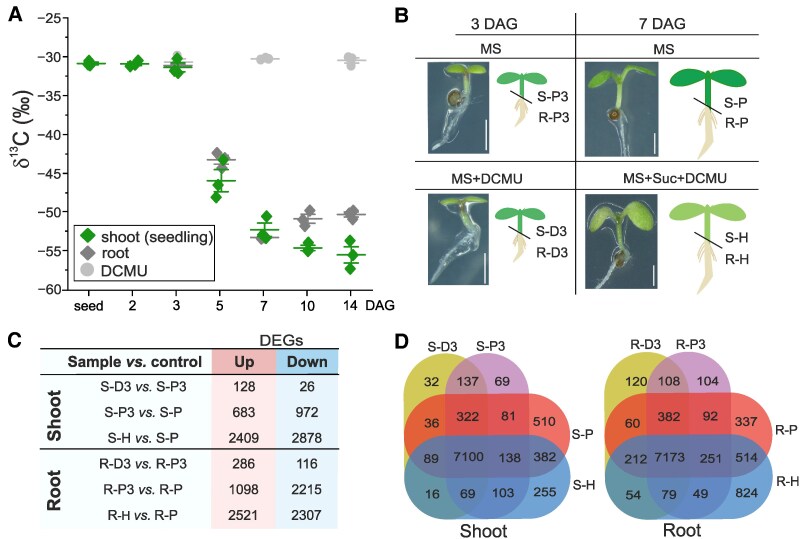
3- and 7-DAG seedlings display distinct gene transcription and H3K27me3 distribution patterns. **A)** Assimilation of atmospheric CO_2_ is initiated after 3 DAG. Plants were cultivated in artificial air (δ^13^C = −40‰). Roots and shoots were analysed separately from 3 DAG. DCMU: photosynthesis-inhibited control. Each point represents a biological replicate; error bars: ±SD; *N* = 3. **B)** Schematic representation of experimental setup. Wild-type seedlings were cultivated on growth medium (MS) supplemented with DCMU (MS + DCMU), or DCMU + 1% sucrose (MS + Suc + DCMU). Scale bar: 1 mm. Tissue samples: S—shoot; R—root; Cultivation conditions: P—photoautotrophic; H—heterotrophic; D—DCMU; 3–3-DAG seedling; 7–7-DAG seedling. The illustrative images of MS seedlings in panel **B)** are also used in Fig. 6G. **C)** Numbers of DEGs in analysed sample comparisons. **D)** Overlap of genes enriched for H3K27me3 (H3K27me3 targets) in the shoot and root samples. DAG—day after germination.

To establish the timepoint of transition from heterotrophy to autotrophy, we first estimated the onset of photosynthetic carbon assimilation in emerging seedlings. To do this, we utilized the difference in CO_2_ isotopic composition between atmospheric air during parental plant growth and seed production (δ ^13^C = −8.5‰) and artificial air applied during seed germination (δ^13^C = −40‰) (Šantrů”ek et al. 2014). Relative decrease of ^13^C showed that photosynthetic CO_2_ assimilation begins after 3 and before 5 days after germination is induced (DAG) (i.e. after the transfer of stratified seeds (SSs) to long-day cultivation) (Fig. 1A). To understand the PRC2-dependent changes connected to the metabolic transition in source and sink tissues, we analysed gene transcription (Fig. 1, B and C, Supplementary Fig. S1A and Supplementary Data Sets S1 to S3) and genome-wide distribution of H3K27me3 (Fig. 1, B and D, Supplementary Fig. S1B—D and Supplementary Data Sets S4 to S6) in the shoots and roots of 3- and 7-DAG seedlings. These timepoints represented steady-state heterotrophic and photoautotrophic metabolic stages of emerging seedlings with similar developmental complexity, that allowed manual dissection of shoot (source) and root (sink) tissues.

To analyse the effect of heterotrophic growth on the plant transcriptome and H3K27me3 distribution, we included plants treated with the photosynthetic inhibitor 3-(3,4-dichlorophenyl)-I,I-dimethylurea (DCMU) that blocks the electron flow from photosystem II to plastoquinone (Rensen 1990) (Fig. 1B). 1% sucrose was added to the DCMU-grown 7-DAG seedlings to retain viability. Principal component analyses (PCA) of the obtained transcriptomic (Supplementary Fig. S1A) and H3K27me3/H3 enrichment data (Supplementary Fig. S1B) confirmed clustering of replicates and separation of shoot and root tissues, as well as 3- and 7-DAG samples. Next, we identified differentially expressed genes (DEGs) between selected pairs of samples (Fig. 1C and Supplementary Data Sets S2 and S3) and genes targeted by H3K27me3 in each sample (Fig. 1D and Supplementary Data Sets S5 and S6). We analysed the relationship between H3K27me3 marking and transcriptional level of the underlying protein-coding genes, focusing on the 7-DAG photoautotrophic samples as a standard growth condition (Supplementary Fig. S2, A—C). High enrichment of H3K27me3 (25% of most highly enriched protein-coding genes) over a large proportion of the gene body (≥70%; “T70” targets) was associated with transcriptional repression, while lower H3K27me3 enrichment or smaller proportion of gene body enriched in H3K27me3 were not predictable indicators of transcriptional repression (Supplementary Fig. S2, A—C). These initial analyses confirmed differences in gene transcription and H3K27me3 distribution between shoot and root tissues in 3- and 7-DAG seedlings.

### Presence of DCMU reduces the deposition of H3K27me3 in 7-DAG seedlings and activates photosynthesis-related genes in the root

**Figure 2.**
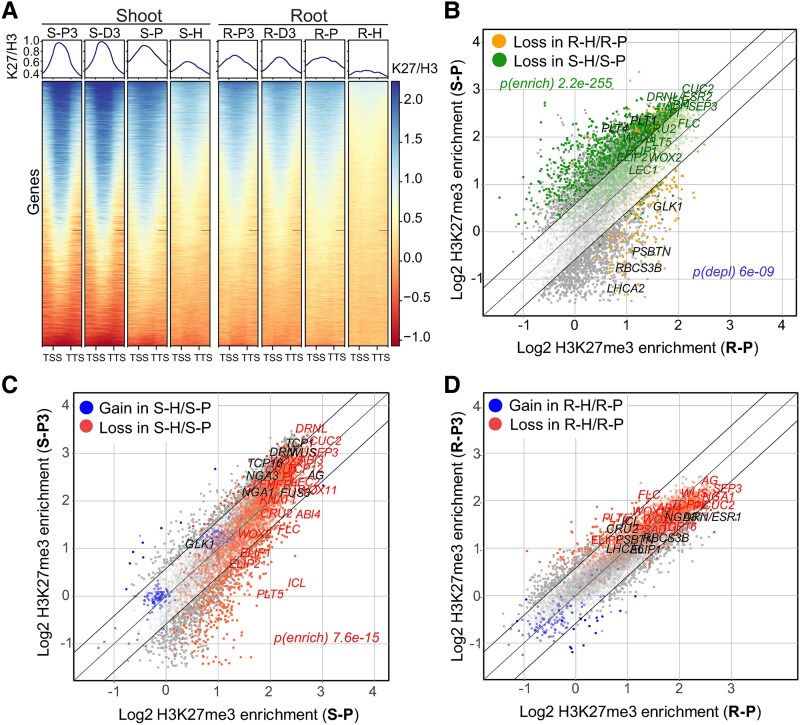
Heterotrophic growth reduces H3K27me3 in 7-DAG tissues. **A)** Log2 H3K27me3/H3 (K27/H3) enrichment in gene bodies (±0.6 kb) of H3K27me3 target genes in wild-type shoot or root. Plotted are H3K27me3 target genes (T70) identified in at least 1 of the shoot samples (“Shoot” panel) or root samples (“Root” panel). Sample labels correspond to Fig. 1B. TSS—transcription start site; TTS—transcription termination site. **B)** H3K27me3/H3 enrichment in H3K27me3 target genes (T70) in 7-DAG shoot (S-P) and root (R-P). Each dot represents a gene; green and orange dots represent genes that lose H3K27me3 in 7-DAG heterotrophic shoot (S-H/S-P) and root (R-H/R-P), resp. *P*-values (hypergeometric test): significance of overlap (enrichment or depletion) between shoot (green) or root (ochre) genes and genes losing H3K27me3 in respective heterotrophic samples. **C)** H3K27me3/H3 enrichment in H3K27me3 target genes (T70) in 3-DAG (S-P3) and 7-DAG (S-P) photoautotrophic shoot. Each dot represents a gene; blue or red dots represent genes that gain or lose H3K27me3, respectively, in 7-DAG heterotrophic compared with photoautotrophic shoot (S-H/S-P). *P*-values (hypergeometric test): significance of overlap between genes that lose H3K27me3 in heterotrophic shoot (S-H/S-P) and genes that gain H3K27me3 between 3 and 7 DAG. **D)** H3K27me3/H3 enrichment in H3K27me3 target genes (T70) in 3-DAG (R-P3) and 7-DAG (R-P) photoautotrophic root. Each dot represents a gene; blue or red dots represent genes that gain or lose H3K27me3, respectively, in 7-DAG heterotrophic compared with photoautotrophic root (R-H/R-P). Outer black diagonal lines in B—D delineate log2 fold-change ± 0.6. DAG—day after germination.

Globally, the enrichment of H3K27me3/H3 in the shoot seemed more concentrated to the gene bodies at 3 DAG than at 7 DAG (Fig. 2A), potentially reflecting spreading of H3K27me3 observed during seedling emergence (Liang et al. 2024). Heterotrophic growth forced by the addition of DCMU had a low impact on H3K27me3 distribution and gene expression in 3-DAG seedlings (Fig. 1C and Supplementary Fig. S1, A and B), indicating limited sensitivity of seedlings to DCMU at this stage. 154 and 402 DEGs were identified in the 3-DAG DCMU-grown shoots and roots compared with respective photoautotrophic tissues. However, at 7 DAG, H3K27me3 was notably reduced (Fig. 1C and Supplementary Fig. S1, A and B). Indeed, 5,287 and 4,828 DEGs were identified in 7-DAG DCMU-grown shoot and root, respectively, compared with photoautotrophic tissues (Fig. 1C and Supplementary Data Sets S2 and S3). The genes upregulated in the heterotrophically (DCMU)-grown 7-DAG shoot were related to oxidative stress, autophagy and catabolic processes, whereas photosynthesis, anabolic metabolism and SA- and JA-mediated responses were downregulated (Supplementary Data Set 2). In the heterotrophic root, the upregulated genes were related to stress responses, light response, photosynthesis, and chloroplasts, while the downregulated genes were related to cell division and root development (Supplementary Data Set 3). Heterotrophically-grown 7-DAG seedlings showed a global reduction of H3K27me3 compared with photoautotrophic 7-DAG seedlings (Fig. 2A and Supplementary Fig. S3, A—B). In the shoot, this was associated with transcriptional increase in the H3K27 demethylases *EARLY FLOWERING 6* and the PRC2 subunit *MEA*, as well as nonsignificant increase of *REF6* and *JUMONJI 32* (*JMJ32*) and the PRC2 subunits *SWN*, *EMF2*, and *MSI1*. However, no similar or consistent transcriptional changes of H3K27 demethylases or PRC2 subunits were found in the root (Supplementary Fig. S3C). Shoot-specific H3K27me3-target genes were more affected by the H3K27me3 loss than root-specific H3K27me3 targets (Fig. 2B, Supplementary Fig. S4, A—D, and Supplementary Data Set 7). Genes marked by high levels of H3K27me3 among the shoot or the root H3K27me3 targets, and genes gaining H3K27me3 between 3 to 7 DAG in the shoot tended to lose H3K27me3 in heterotrophically-grown seedlings (Fig. 2, C and D, and Supplementary Data Set 8). Genes that lost H3K27me3 in heterotrophically-grown shoots were enriched in developmental and transcription-related processes, whereas genes with unchanged H3K27me3 are mostly related to metabolic processes (Supplementary Fig. S4E and Supplementary Data Set 8). Importantly, there was a limited overlap between genes that lost H3K27me3 and genes that were upregulated in heterotrophic shoot compared with photoautotrophic shoot (Supplementary Fig. S4F), indicating uncoupled effects of heterotrophic growth on H3K27me3 and gene transcription.

### Between 3 and 7 DAG, developmental and metabolic genes become repressed in the shoot, while photosynthesis-related genes become repressed in the root

**Figure 3.**
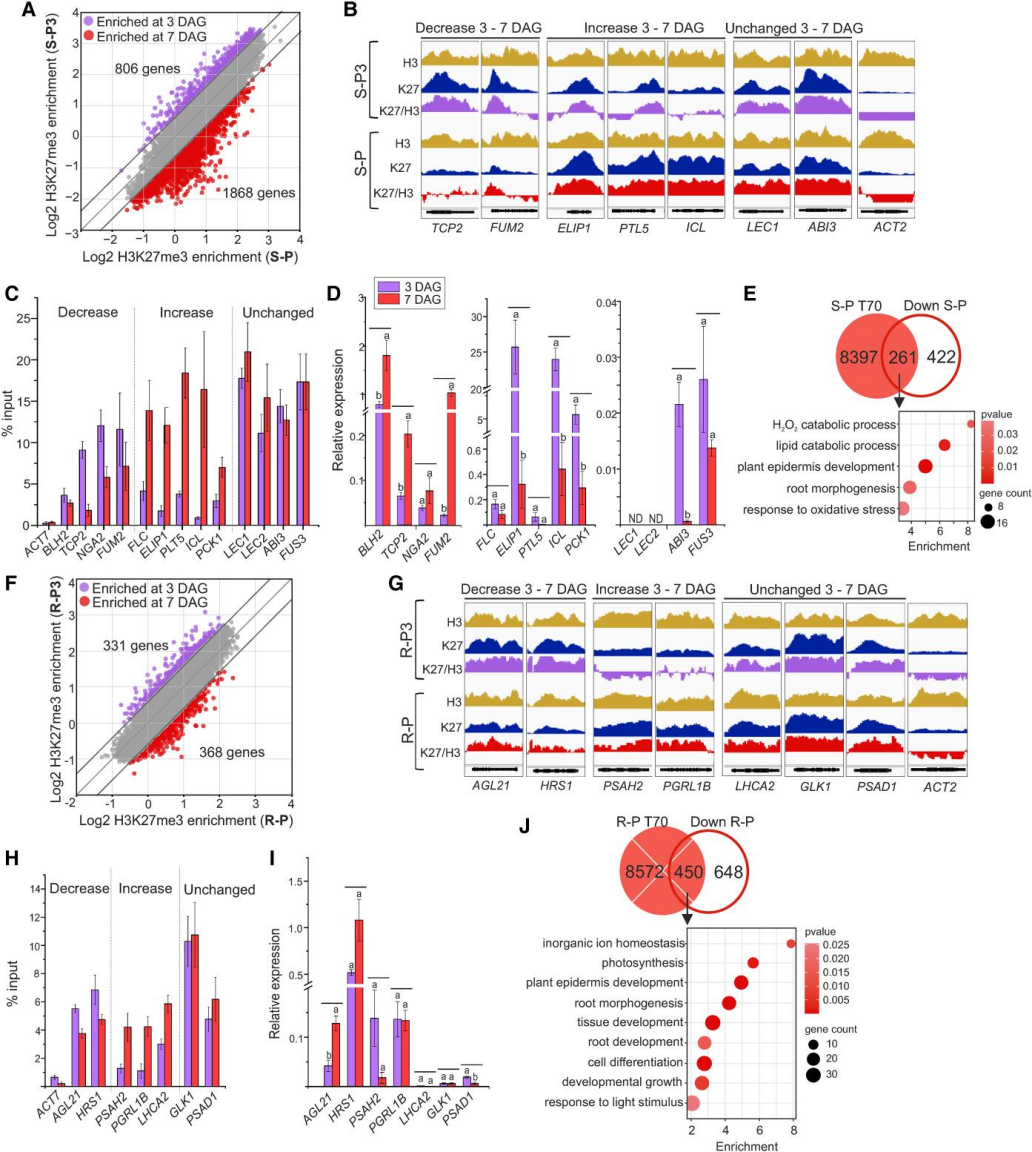
H3K27me3 and transcriptional reprogramming in shoot and root tissues between 3 and 7 DAG. **A)** and **F)** H3K27me3/H3 enrichment in target genes in 3-DAG and 7-DAG photoautotrophic shoot (S-P3, S-P; resp.) **A)** or root (R-P3, R-P; resp.) **F)**. Genes enriched for H3K27me3/H3 in at least 1 sample are displayed. Outer black diagonal lines in B—D delineate log2 fold-change ± 0.6. **B)** and **G)** Genome browser display of selected genes showing the distribution of H3, H3K27me3 (“K27”), and H3K27me3/H3 (“K27/H3”) in 3-DAG and 7-DAG wild-type shoot **B)** or root **G)**. The *Y*-axis scale of the respective H3, K27 and K27/H3 tracks compared at 3 DAG and 7 DAG is identical. *ACT2*, a non-PRC2-target gene, serves as a negative control. **C)** and **H)** ChIP-qPCR confirmation of ChIP-seq. **C)** genes with decreased (*BLH2, TCP2, NGA2, FUM2*), increased (*FLC, ELIP1, PLT5, ICL, PCK1*), and unchanged (*LEC1, LEC2, ABI3, FUS3*) H3K27me3 between 3- and 7-DAG shoot. **H)** genes with decreased (*AGL21* and *HRS1*), increased (*PSAH2* and *PGRL1B*), or unchanged (*LHCA2, GLK1* and *PSAD1*) H3K27me3 between 3- and 7-DAG root. *ACT7* serves as negative control with no H3K27me3 enrichment. Bars: mean ± SD; *N* = 3 technical replicates. **D)** and **I)** Transcription (RT-qPCR) of genes analysed in **C)** and **H)**, respectively. Bars: mean ± SD; *N* = 3 biological replicates. Letters above bars: statistical significance levels at *P* < 0.01; Student’s *t* test. ND—not detected. **E)** and **J)** Gene ontology enrichment of 7-DAG H3K27me3 target genes (T70) transcriptionally downregulated from 3 to 7-DAG in the shoot **E)** or root **J)**. S-P: 7-DAG shoot; R-P: 7-DAG root. BP categories are shown; GO display cutoff: fold enrichment > 1.5; p(Bonferroni) < 0.05. DAG—day after germination.

To understand the changes that occur between 3 and 7 DAG and may thus contribute to the metabolic reprogramming in emerging seedlings, we analysed H3K27me3 distribution and gene expression at these 2 time points in both shoots and roots. To identify genes that require PRC2 for their repression, we first focused on genes that were downregulated and gained H3K27me3 between 3 and 7 DAG. 683 genes were downregulated between 3 and 7 DAG in the shoot (Fig. 1C, Supplementary Data Set 2). These genes were enriched for plant-type cell wall organization, lipid catabolism and oxidation-reduction processes (Supplementary Data Set 2). 1,868 PRC2 target genes gained H3K27me3 in the shoot (Fig. 3A and Supplementary Data Set 9). 97 of these genes are TF genes (*TFs*) enriched for phyllotactic patterning and negative regulation of flowering, and include the *APETALA 2* (*AP2*) *TFs PLETHORA 3* (*PLT3*), *PLT5/EMK*, *PLT7*, the MADS TF *AGL15*, or the flowering repressors *FLOWERING LOCUS C* (*FLC*), *MADS AFFECTING FLOWERING 4* (*MAF4*) and *MAF5*. Other genes that gain H3K27me3 include for instance the auxin transporter *PIN-FORMED 1* (*PIN1*), thylakoid-bound *EARLY LIGHT-INDUCIBLE PROTEIN 1* (*ELIP1*) and *ELIP2*, the glyoxylate cycle enzyme-encoding gene *ICL*, the gluconeogenesis-related enzyme-encoding gene *PHOSPHOENOLPYRUVATE CARBOXYKINASE 1* (*PCK1*) and genes encoding enzymes of reserve lipid catabolism, such as *OIL BODY LIPASE 1* (*ATOBL1*) (Fig. 3, B and C, Supplementary Fig. S5A and Supplementary Data Set 9). This is reflected in decreasing transcription of these genes, including *FLC*, *ELIP1*, *PLT5*, *ICL*, and *PCK1* (Fig. 3D). 261 genes marked by H3K27me3 at 7-DAG (T70 genes) were downregulated between 3 and 7-DAG (Fig. 3E and Supplementary Data Set 10). These genes were enriched for biological processes related to epidermis and root development, lipid and hydrogen peroxide catabolism, and oxidative stress response (Fig. 3E). Notably, the *LAFL* genes were already marked by H3K27me3 and transcriptionally repressed at 3 DAG, but further reduction in *ABI3* transcription could be detected between 3 and 7 DAG in the shoot (Fig. 3, B—D). In contrast, 972 genes were upregulated in the shoot between 3 and 7 DAG (Fig. 1C and Supplementary Data Set 2), of which 375 were marked by H3K27me3 at 3 DAG (Supplementary Fig. S5B and Supplementary Data Set 10). These genes were enriched for abiotic and biotic stress responses, hormone signaling including SA and JA, and glucosinolate metabolism (Supplementary Fig. S5B). H3K27me3 marking was reduced over 806 genes between 3 and 7 DAG (Fig. 3A and Supplementary Data Set 9), of which 89 were *TFs*. These included lateral boundary and shoot organ development *TFs*, such as *NGATHA 2* (*NGA2*) and *NGA3*, TALE homeodomain *TFs BELL1*, *BEL1-LIKE HOMEODOMAIN 2* (*BLH2*), *BLH4*, *KNOTTED1-LIKE HOMEOBOX GENE 4* (*KNAT4*), MYB domain *TFs LATERAL ORGAN FUSION 1* (*LOF1*) and *LOF2*, TEOSINTE BRANCHED 1, CYCLOIDEA and PCF domain (TCP) *TFs* including *TCP2* (Fig. 3, B—D, Supplementary Fig. S5A and Supplementary Data Set 9).

Following the approach taken in the shoot, we analysed the differences in H3K27me3 and in the transcriptome between 3- and 7-DAG roots. 1,098 genes were downregulated between 3 and 7-DAG in the root (Fig. 1C and Supplementary Data Set 3). These genes were significantly enriched for root morphogenesis, light response and photosynthesis. 368 genes gained H3K27me3 in the root (Fig. 3, F—I, Supplementary Fig. S5C and Supplementary Data Set 11). 450 genes marked by H3K27me3 at 7 DAG were downregulated between 3 and 7 DAG, and these were enriched for inorganic ion homeostasis, photosynthesis and light response, as well as root and epidermis development (Fig. 3J and Supplementary Data Set 12). Notably, several photosynthesis and light response-related genes were already marked by H3K27me3 at 3 DAG (Fig. 3, G—I), suggesting that repression of these genes may be an ongoing process initiated before 3 DAG. In contrast, 2,215 genes activated in the root from 3 to 7 DAG were mainly enriched in responses to biotic and abiotic stimuli or stress (Supplementary Data Set 3). 795 genes marked by H3K27me3 at 3 DAG were upregulated between 3 and 7 DAG (Supplementary Fig. S5D and Supplementary Data Set 12). Similar to the shoot, these genes were enriched in biotic and abiotic stress responses.

Overall, in both shoots and roots, biotic and abiotic stress response genes were released from H3K27me3-mediated repression and activated between 3 and 7 DAG. In the shoot, genes associated with embryo maturation and biosynthesis of embryonic storage compounds were already marked by H3K27me3 and transcriptionally repressed at 3 DAG. Genes involved in root development, seed germination, catabolism of storage lipids, the glyoxylate cycle, and gluconeogenesis, were repressed between 3 and 7 DAG. In the root, photosynthesis and light response-related genes were found among the genes that gained H3K27me3 between 3 and 7 DAG and were downregulated during seedling establishment.

### PRC2 represses exogenous sugar-induced accumulation of storage lipids and promotes photoautotrophic growth

**Figure 4.**
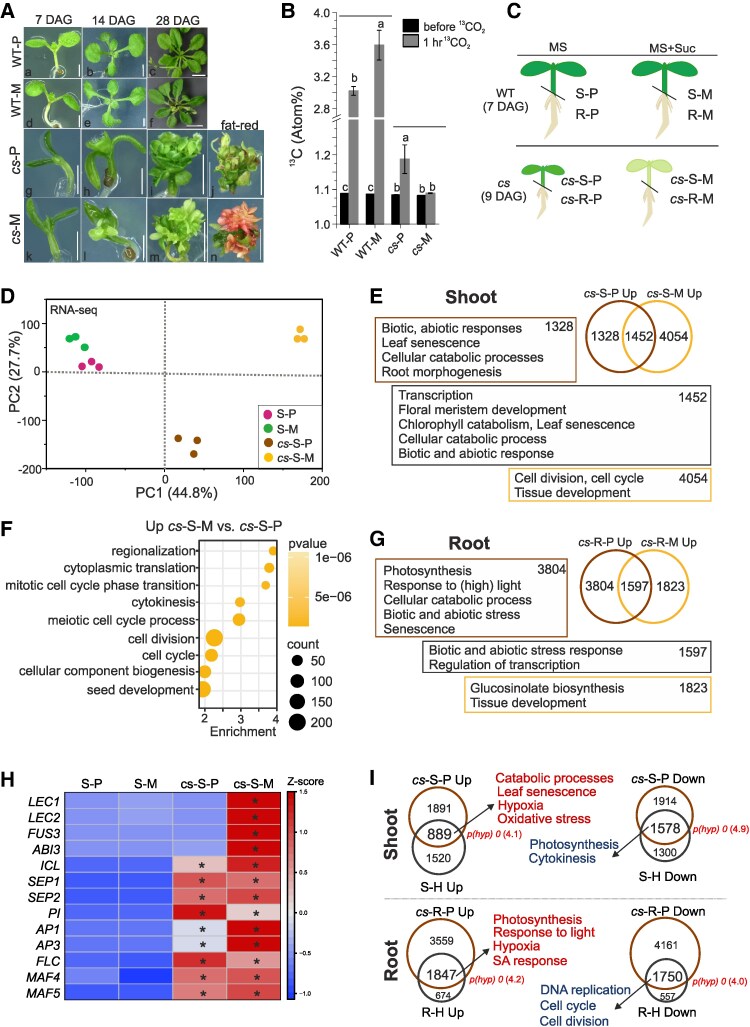
PRC2 is required for photoautotrophic growth. **A)** Wild type (WT; a-f) and *clf swn* (*cs*; g-n) plants cultivated in photoautotrophic (P) and mixotrophic (M) conditions for 7, 14 and 28 days. Sudan Red 7B (fat- red) staining (j, n) is used to detect embryonic lipids. Scale bar = 1 mm (a, d, g, h, k, l), 2 mm (i, j, m, n), 5 mm (b, e), 1 cm (c, f). **B)**  ^13^CO_2_ content in the shoot before and after 1-hr cultivation in ^13^CO_2_-enriched air. WT and *cs* shoots of 10-DAG seedlings cultivated in photoautotrophic (P) and mixotrophic (M) growth conditions are shown. Approximately 1.1 atom% of ^13^C in all WT and *cs* represents natural abundance of ^13^CO_2_ in atmospheric air. Bars: mean ± SD; *N* = 3 biological replicates. Letters above bars: *P* < 0.05; two-way ANOVA with Bonferroni post hoc test. **C)** Experimental setup of transcriptome analyses of seedlings 5 to 6 DAG. 9-DAG *cs* and 7-DAG WT is used to account for the delay in *cs* germination. S—shoot; R—root; P—photoautotrophic; M—mixotrophic; MS—½ Murashige and Skoog medium; suc—sucrose. **D)** RNA-Seq PCA plot: RPKM values of all genes. **E)** Schematic representation of genes and enriched GO biological processes upregulated in *cs* photoautotrophic (*cs*-S-P) and mixotrophic (*cs*-S-M) shoots compared with respective WT shoots (S-P and S-M). GO summary cutoff: p(Bonferroni) < 0.05. **F)** Biological processes upregulated in mixotrophic (*cs*-S-M) compared with photoautotrophic (*cs*-S-P) *cs* shoot. GO summary cutoff: p(Bonferroni) < 1e-06. **G)** Schematic representation of genes and enriched GO biological processes upregulated in *cs* photoautotrophic (*cs*-R-P) and mixotrophic (*cs*-R-M) roots compared with respective WT roots (R-P and R-M). GO display cutoff: p(Bonferroni) < 0.05. **H)** Expression of embryo and flower development genes in WT and *cs*. *Z*-scored RPKM; asterisks (*): significantly different transcription related to corresponding WT (FDR < 0.05, DESeq2). **I)** Photoautotrophic *cs* seedlings resemble heterotrophic WT seedlings. p(hyp): *P*-value—hypergeometric test of enrichment; ratio of observed/expected indicated in brackets. Summary of GO biological processes enriched among genes commonly dysregulated in photoautotrophic *cs* and DCMU-treated WT tissues. GO summary cutoff: fold enrichment > 3; p(Bonferroni) < 0.05. Full GO graphs are shown in Supplementary Fig. S8. DAG—day after germination.

Embryonic depletion of FIE (Bouyer et al. 2011) or CLF and SWN (Supplementary Fig. S6, A—C) does not impede embryo or seed development, but it significantly affects postembryonic development (Fig. 4A). Germination of *cs* seeds was delayed by 2 to 3 days regardless of growth conditions (Supplementary Fig. S6D) and seedlings developed cotyledon-like pale green shoot structures that accumulated TAGs (Fig. 4A—“*cs*-M” and Supplementary Fig. S6, E and F) (Chanvivattana et al. 2004; Aichinger et al. 2009; Bouyer et al. 2011). Importantly, the described developmental phenotypes and the ectopic accumulation of storage lipids was conditioned by continuous presence of sucrose in the cultivation medium since germination, or its supply before 3 DAG (Supplementary Fig. S6, G and H). In contrast, in the absence of exogenous sucrose or its supply after 3 DAG, green vegetative *cs* plantlets developed that did not accumulate TAGs (Fig. 4A—“*cs*-P” and Supplementary Fig. S6, E—H). Unlike WT plants, sucrose-grown *cs* plants failed to assimilate atmospheric CO_2_ (Fig. 4B) but accumulated biomass (Supplementary Fig. S6I), indicating heterotrophic growth. In contrast, *cs* plants grown without external sucrose assimilated atmospheric CO_2_ (Fig. 4B) and accumulated biomass (Supplementary Fig. S6I), indicating photoautotrophic growth. Notably, the assimilation and growth rate in photoautotrophic *cs* plants was significantly lower than in WT plants and they exhibited embryonic flower-like phenotypes and homeotic defects (Fig. 4A—“*cs*-P”). These results indicated that PRC2 activity in the initial phases of seedling emergence is essential to repress pathways that direct sucrose towards accumulation of storage lipids and to stimulate photoautotrophic growth.

To identify transcriptional patterns associated with phenotypic differences in *cs* (Fig. 4A), we analysed the transcriptome of photoautotrophic and mixotrophic WT and *cs* shoots and roots 5 to 6 days after the seeds had germinated (i.e. after the switch to photoautotrophy—Fig. 1A). To account for the delayed germination in *cs*, 7-DAG WT and 9-DAG *cs* plants were analysed (Fig. 4C, Supplementary Fig. S7A and Supplementary Data Set 13). Consistent with the observed differences in developmental phenotypes (Fig. 4A), sucrose enhanced the separation of WT and *cs* shoot samples, and the transcriptome of WT was less affected by the presence of sucrose than that of *cs* (Fig. 4D). This was reflected in 739 sucrose-induced DEGs in WT but 10,896 in *cs* shoot (Supplementary Fig. S7B and Supplementary Data Set 13). A similar effect was observed in the root tissues (Supplementary Fig. S7A and Supplementary Data Set 14), corroborating massive sucrose-induced transcriptional reprogramming in *cs*.

To understand the exogenous sugar-dependent or independent effects of *cs* on transcription, we compared the DEGs in *cs* related to WT in mixotrophic or photoautotrophic shoots (Fig. 4, E and F, Supplementary Fig. S7, C and E, and Supplementary Data Sets S13 and S15). In *cs* shoot (Fig. 4E), 1,452 genes related to transcription, floral meristem, catabolic processes, biotic and abiotic responses and senescence were upregulated in both growth conditions. Similar processes were also enriched among the 1,328 genes that were specifically upregulated in the photoautotrophic shoot. 4,054 genes that were only upregulated in the mixotrophic *cs* shoot were related to the cell cycle and tissue development. Conversely, photosynthesis and light response were downregulated in the *cs* shoot under both growth conditions (Supplementary Fig. S7C). Genes related to cell division and seed development were upregulated in the mixotrophic compared with photoautotrophic *cs* shoot (Fig. 4F), while biotic and abiotic stress responses and light responses were upregulated in photoautotrophic *cs* shoot (Supplementary Fig. S7E). Importantly, upregulation of the *LAFLs*—*LEC1*, *LEC2*, and *FUS3*—was limited to mixotrophic *cs* (Fig. 4H, Supplementary Fig. S7F), as was the accumulation of abscisic acid (Supplementary Fig. S7G). In contrast, metabolic genes involved in early seedling establishment, including *ICL*, were upregulated regardless of growth conditions. Similarly, other known PRC2-target genes, including the floral identity MADS-box TF genes *SEPALLATA 1* (*SEP1*), *SEP2*, *PISTILLATA* (*PI*), *APETALA 1* (*AP1*), *AP2* or the floral repressor *FLC*, *MAF4*, or *MAF5*, were upregulated independently of sucrose, indicating an uncoupled effect of sucrose on the regulatory networks controlling different developmental pathways (Fig. 4H). In the *cs* root, genes connected to biotic and abiotic stress were upregulated in both conditions, while photosynthesis and light response genes were upregulated mainly in the photoautotrophic root (Fig. 4G). At the same time, root morphogenesis and growth-related genes were downregulated, especially in photoautotrophic *cs* roots (Supplementary Fig. S7D). Notably, the transcriptomic changes in photoautotrophic *cs* resembled heterotrophically-grown WT plants (Fig. 4I and Supplementary Fig. S8, A—E, and Supplementary Data Set 16), marked by elevated senescence- and stress-related gene expression in the shoot, induction of photosynthesis and light-response genes in the root, and by general suppression of cell division- and growth-related genes. Collectively, PRC2 represses sucrose-induced upregulation of genes related to seed development, including *LAFL*, ectopic accumulation of storage lipids, and heterotrophic growth. In the absence of exogenous sucrose, PRC2 is not essential for the stable transition to photoautotrophy, but promotes photosynthetic carbon assimilation and vegetative growth.

### Shoot of PRC2-depleted seedlings displays transcriptional and metabolic signatures of developing seeds and early emerging seedlings

**Figure 5.**
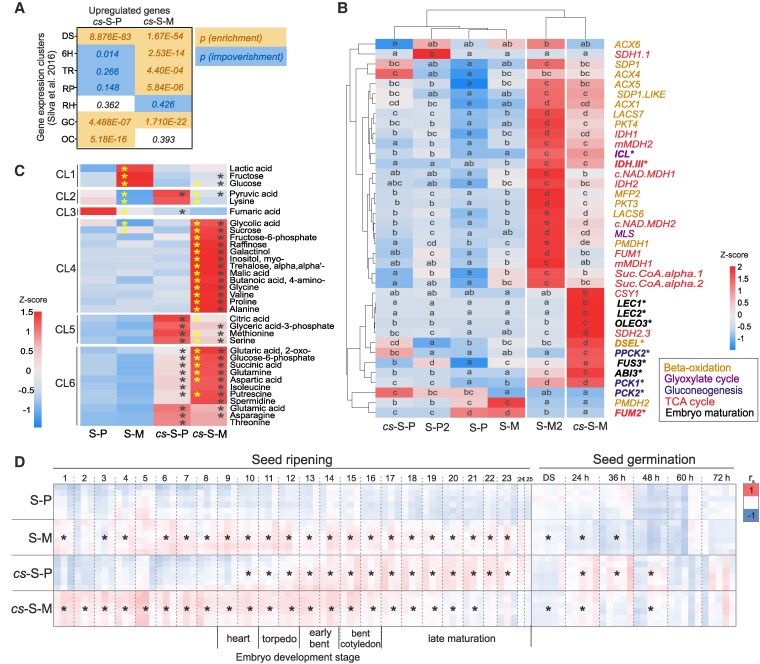
The transcriptome and primary metabolome of PRC2-depleted *clf swn* (*cs*) seedlings resembles embryo maturation and seed germination developmental stages. **A)** Comparison of DEGs in 9-DAG photoautotrophic (*cs*-S-P) and mixotrophic (*cs*-S-M) *cs* seedling shoots to genes transcribed in dry seed and seedling establishment stages (Silva et al. 2016). *P*-values of hypergeometric tests for gene set overlaps: enrichment and impoverishment compared to overlaps expected by chance are distinguished. DS—dry seed, 6H—six hours of imbibition, TR—testa rupture, RP—radicle protrusion, RH—root hair emergence stage, GC—greening cotyledon stage, OC—open cotyledons stage (cf. full dataset comparison in Supplementary Fig. S9, B and C). **B)** RT-qPCR-based determinations of transcript abundances of genes involved in embryo maturation and metabolic pathways marking seedling emergence in photoautotrophic (S-P2 and S-P) and mixotrophic (S-M2 and S-M) WT shoot at 2- and 7-DAG and photoautotrophic (*cs*-S-P) and mixotrophic (*cs*-S-M) *cs* shoot at 9-DAG. Mutant sampling was delayed to adjust mutant development to 7-DAG WT. The heatmap represents z-score normalized relative expression mean of 3 biological replicates. Letters (a—e): statistical significance at *P* < 0.05, based on a two-way ANOVA. **C)** Heatmap of selected relative metabolite abundances in WT photoautotrophic (S-P) and mixotrophic (S-M) shoots of WT seedlings compared with respective *cs* shoot samples *cs*-S-P and *cs*-S-M. *Z*-score normalized means are represented; *N* = 5 biological replicates. Asterisks (*): statistical significance at *P* < 0.05 (adjusted) based on ANOVA and Tukey's HSD test. Black asterisks indicate significant difference among genotypes (*cs*-S-P vs. S-P and *cs*-S-M vs. S-M), yellow asterisks indicate significant differences induced by mixotrophy (S-M vs. S-P and *cs*-S-M vs. *cs*-S-P). Clusters (CL) 1 to 6 correspond to Supplementary Fig. S9F, i.e. the complete heatmap of all 85 metabolites. **D)** Spearman correlation between the primary metabolomes of photoautotrophic (P) and mixotrophic (M) WT and *cs* shoot samples and samples representing 25 stages of seed maturation, dry seeds (DS) and 5 stages of seed germination sampled at 24, 36, 48, 60, and 72 h after imbibition(Ginsawaeng et al. 2021). Metabolite data were maximum-scaled per metabolite and dataset to a 0 to 100 numerical range. Negative correlation (−1 minimum correlation coefficient, blue), noncorrelated (0, white), and positive correlation (+1 maximum correlation coefficient, red). Asterisks (*): significant differences between replicates of correlation coefficients (Student’s *t*-test, *P* < 0.05, 2-tailed, heteroscedastic) tested against the respective WT photoautotrophic (S-P) replicates. DAG—day after germination.

Transcriptional changes in *cs* indicated activation of metabolic programs associated with seed maturation, germination and early seedling establishment. Therefore, we compared genes upregulated in *cs* shoot with previously published clusters of genes peaking at different stages of Arabidopsis seed-to-seedling transition (Silva et al. 2016) (Fig. 5A and Supplementary Fig. S9, A—C). The photoautotrophic *cs* shoot resembled dry seeds and early greening seedlings, whereas mixotrophic *cs* shoot showed highest similarity to dry and early germinating seeds (Fig. 5A). Next, we analysed the transcription of key embryo maturation and seedling establishment metabolic genes in WT at the early greening stage (2 DAG) and after photoautotrophic transition (7 DAG), and compared this to 9-DAG *cs*, using photoautotrophic and mixotrophic seedling shoots (Fig. 5B). Among the analyzed samples, 9-DAG *cs* most closely resembled 2-DAG WT, particularly in mixotrophy, that strongly promoted transcription of β-oxidation, glyoxylate metabolism and TCA cycle genes in both the genotypes, especially in WT. Importantly, the *LAFL* TFs were not activated by exogenous sucrose in 2-DAG WT, indicating that they were stably repressed at this stage. In *cs*, transcription of *LAFLs* was comparable to 2-DAG WT levels in photoautotrophic state but transcripts strongly accumulated in mixotrophic sample, i.e. upon supplementation by external sucrose (Fig. 5B).

To understand whether these changes are reflected in the metabolome, we analyzed primary metabolites in 7-DAG WT and 9-DAG *cs* shoots under photoautotrophic and mixotrophic conditions (Fig. 5C and Supplementary Fig. S9, D—F, and Supplementary Data Set 17). PCA and contribution plot of the primary metabolome indicated that the differences in genotypes and supplementation of sucrose were the main sources of variation (Supplementary Fig. S9, D and E). Amino acids and organic acids contributed highly to the differences between genotypes. Sucrose, fructose (Fru) and glucose (Glc) among other metabolites distinguished photoautotrophy from mixotrophy (Supplementary Fig. S9E). The 85 identified primary metabolites were grouped into 6 clusters (CL1—CL6, Supplementary Fig. S9F), highlighting the differences between genotypes and the differential effects of exogenous sucrose on WT or *cs*. In particular, metabolization of sucrose provided to mixotrophic shoot differed between WT and *cs* shoots. WT accumulated Fru and Glc (CL1) indicating uptake and a limited metabolization of sucrose, e.g. by invertase-catalyzed cleavage. In contrast, *cs* phosphorylated Glc and Fru and metabolized hexose-phosphates further (CL4, CL5, and CL6). Glc6P and Fru6P accumulated in *cs* together with glyceric acid-3P and intermediates of the TCA cycle, including malic acid, citric acid, 2-oxoglutaric acid and succinic acid (Supplementary Fig. S9F). Catabolism of supplied sucrose extended up to and beyond the TCA and was associated with anabolic accumulation of most proteinogenic amino acids in photoautotrophic and mixotrophic *cs* (CL5 and CL6). In contrast to WT or photoautotrophic *cs*, mixotrophic *cs* accumulated raffinose, galactinol and myo-inositol, metabolites of the raffinose family oligosaccharide biosynthesis pathway, as well as salicylic acid, proline, 4-aminobutyric acid (GABA), and trehalose (CL4). Similarly, putrescine and spermidine accumulated most in mixotrophic *cs* (CL6). The presence of these marker metabolites indicated pronounced physicochemical stress (Dempsey et al. 2011; Zandalinas et al. 2022). Mixotrophic *cs* accumulated TAGs (Fig. 4A and Supplementary Fig. S6, E—H). This process is expected to consume acetyl-CoA for FA biosynthesis and to redirect acetyl-CoA from metabolization by the TCA cycle. Indeed, citric acid (CL5), which is synthesized by citrate synthase from acetyl-CoA and oxaloacetate at the entry point of the TCA cycle was the only intermediate of the TCA cycle that accumulated less in mixotrophic *cs* than in photoautotrophic *cs*.

Next, we compared the metabolome of the *cs* shoot with different stages of seed development, maturation, and germination. We profiled the metabolome of 25 WT seed developmental stages, corresponding to embryo morphogenesis stages up to the late embryo maturation (Supplementary Fig. S10A and Supplementary Data Set 18). In addition, we analyzed previously published data of primary metabolites from developmental series of germinating seeds (Ginsawaeng et al. 2021). 53 metabolites were robustly identified in all samples and overlapped with the current study (Supplementary Data Set 19). Weighted correlation network analysis (WGCNA) indicated that relative amounts of amino acids and organic acids are depleted in dry seeds but accumulate during germination (Supplementary Fig. S10, B—E, and Supplementary Data Set 20), which was consistent with earlier studies (Fait et al. 2006; Silva et al. 2017). Next, we compared the abundance patterns of samples from the current study to the chosen reference profiles by nonparametric correlation across the 53 commonly detected metabolites (Fig. 5D and Supplementary Data Set 21). The primary metabolome of the photoautotrophic WT shoot was largely uncorrelated or correlated negatively with the seed maturation or germination developmental samples. In contrast, the samples from photoautotrophic *cs* shoot correlated positively with the late maturation stages of embryogenesis and seedlings at 24 to 48 h after germination. Exogenous sucrose in WT induced metabolic changes that enhanced correlations to the early to intermediate stages of seed development. The positive correlation with early seed developmental stages was further enhanced in *cs* samples (Fig. 5D).

In summary, *cs* seedlings exhibited transcriptional and metabolic characteristics of maturing embryos, germinating seeds, and early seedling establishment stages. In particular, transcripts of genes involved in the degradation of storage lipids, i.e. FA β-oxidation, the glyoxylate cycle, gluconeogenesis and the TCA cycle accumulated. These transcriptional similarities are associated with the metabolic signatures of late embryogenesis and germinating seeds that are retained in *cs* seedlings. Application of exogenous sucrose promotes metabolic patterns of early developing embryos in *cs* seedlings beyond the WT and induces a complex metabolic stress response.

### Two phases of PRC2 repression are required for the establishment of photoautotrophic seedling

**Figure 6.**
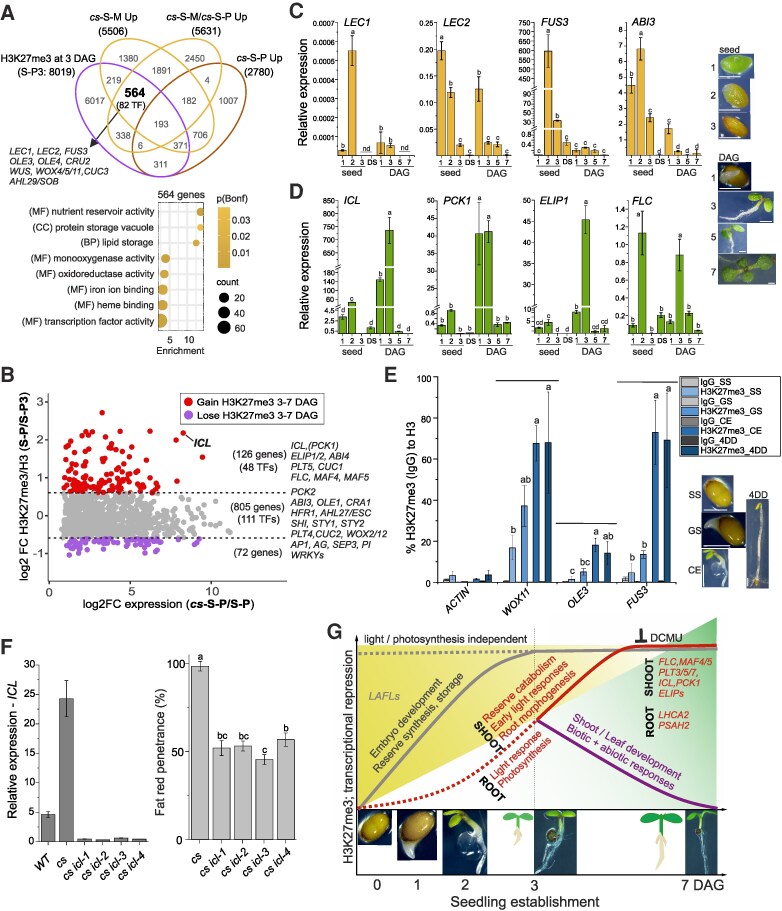
PRC2 coordinates developmental and metabolic reprogramming in several phases of seedling establishment. **A)** Identification of H3K27me3-target genes that contribute to reversal to lipid accumulating (embryo maturation) phase in mixotrophically-grown *clf swn* seedlings. Comparison of gene sets marked by H3K27me3 at 3-DAG (S-P3), genes upregulated in mixotrophic *cs* shoot compared with respective WT (*cs*-S-M Up), genes upregulated in photoautotrophic *cs* shoot compared with respective WT (*cs*-S-P Up) and gene upregulated in mixotrophic compared with photoautotrophic *cs* shoot (*cs*-S-M/*cs*-S-P Up). Selected key regulators of embryo development are highlighted among 564 protein-coding genes. GO analysis of 564 genes that contribute to the metabolic/developmental reversal. GO display cutoff: fold enrichment > 1.5; p(Bonferroni) < 0.05. **B)** Identification of genes marked by H3K27me3 at 3 and/or 7-DAG that contribute to the *cs* shoot phenotype during photoautotrophic growth. Y-axis: fold-change (FC) H3K27me3/H3 enrichment in 7-DAG (S-P) compared with 3-DAG (S-P3) WT shoot; X-axis: FC expression of DEGs upregulated in *cs* (*cs*-S-P) compared with WT (S-P) photoautotrophic shoot. Expression log2 FC cutoff is 0.6. Each dot represents a gene: 126 genes (48 transcription factor genes—TFs) gain H3K27me3 between 3- and 7-DAG WT shoot and are upregulated in *cs*; 72 genes that lose H3K27me3 between 3- and 7-DAG WT shoot and are upregulated in *cs*; 805 genes (111 TFs) with unchanged levels of H3K27me3 between 3- and 7-DAG WT shoot are upregulated in *cs*. H3K27me3/H3 log2 FC cut-off is 0.6. Selected key developmental or metabolic regulators are highlighted. **C)** Expression of genes marked with H3K27me3 by 3 DAG. **D)** Expression of genes gaining H3K27me3 between 3 and 7 DAG. **C)** and **D)** qRT-PCR analysis of selected gene transcription in 3 seed development stages (“seed”: 1—mature green, 2—mature yellowing, 3—mature desiccating), dry seeds (DS) and 4 stages of seedling germination (DAG: 1, 3, 5 and 7). Representative seeds/seedlings are shown on the right. Scale bar = 0.5 mm (seed); 1 mm (seedling). Bars: mean ± SD; *N* = 3 biological replicates. Letters above bars: statistical significance at *P* < 0.05; one-way ANOVA with Bonferroni post hoc test. ND—not detected. **E)** H3K27me3 deposition during seed germination is independent of active photosynthesis. ChIP-qPCR of H3K27me3 enrichment at 4 stages of seedling establishment before the onset of photosynthesis: SS, germinated seed (GS), cotyledon emergence (CE) and 4-DAG dark-grown seedling. Bars: mean ± SD; *N* = 3 biological replicates. Letters above bars: statistical significance at *P* < 0.05; one-way ANOVA with Bonferroni post hoc test. Representative images of developmental stages analysed are depicted on the right. Scale bar = 0.5 mm (seed); 1 mm (seedling). **F)** CRISPR-Cas9 mutagenesis of *ICL* in *clf swn* genetic background (*cs icl*) limits the embryonic reversal in *cs*. RT-qPCR of *ICL* expression (left). Bars: mean ± SD; *N* = 3 technical replicates. Penetrance of positive fat-red staining phenotype (right) in *cs (icl)* seedlings grown in the presence of 1% sucrose. Bars: mean ± SD; *N* = 3 biological replicates (20 to 30 *cs icl* seedlings/replicate). Letters above bars: statistical significance at *P* < 0.05; one-way ANOVA with Bonferroni post hoc test. **G)** Summary of PRC2 contribution to gene repression at different phases of seedling establishment. Backgrounds represent seed (heterotrophic—yellow) to seedling (photoautotrophic—green) transition. Gray and red lines/font represent increase in H3K27me3 (transcriptional repression) before 3-DAG and between 3 and 7 DAG, respectively. Purple line/font represent decrease in H3K27me3 (transcriptional activation) between 3 and 7 DAG. Representative developmental seed and seedling stages are shown in the bottom; these illustrative images are also used in panel B and in Fig. 1B. DAG—day after germination.

Based on the preceding analyses (Fig. 3, A—D, Fig. 5B and Supplementary Fig. S6H), we hypothesized that in the time window of 3 DAG, PRC2 represses sucrose induced ectopic activation of *LAFL* genes and pathways that direct sucrose towards TAG biosynthesis. To identify the underlying genes, we compared genes marked by H3K27me3 at 3-DAG with genes upregulated in mixotrophic, but not in photoautotrophic, *cs* shoot (Fig. 6A). We identified 564 genes enriched for lipid storage and associated GO categories. Among these were 82 *TFs*, including *LEC1*, *LEC2* and *FUS3* (Fig. 6A and Supplementary Data Set 22). Next, we asked which pathways require PRC2 for their repression in order for the vegetative (photoautotrophic) state to be established. 805 genes marked by H3K27me3 in 3- and 7-DAG WT shoot were upregulated in photoautotrophic *cs* compared with WT shoot (Fig. 6B and Supplementary Data Set 23). These genes were involved in seed, root, or flower development, auxin biosynthesis, oxidative stress response and biotic defence responses (Supplementary Data Set 23). Among them, 111 genes encoded TFs, including key transcriptional regulators of these processes (Fig. 6B). 126 (48 TFs) genes upregulated in photoautotrophic *cs* shoot gained H3K27me3 between 3 and 7-DAG in WT (Fig. 6B and Supplementary Data Set 23). This group of genes included genes related to the glyoxylate cycle (*ICL*) and gluconeogenesis (*PCK1*), seed development (*ABI4*, *LEAs*), early light response (*ELIP1*, *ELIP2*), root (*PLT5*) and shoot (*CUC1*) development and the flowering repressors *FLC*, *MAF4*, and *MAF5*. Hence, PRC2 transcriptionally represses distinct groups of genes during the seed-to-seedling transition—genes repressed by 3 DAG and potentially reactivated by exogenous sucrose, and genes repressed between 3 and 7 DAG. Accordingly, the transcription *LAFL* genes, as representatives of the genes repressed by 3 DAG, peaked during embryo maturation and at 1 DAG, but was low at 3 DAG (Fig. 6C). In contrast, transcription of seed germination metabolic genes, as representative of genes gaining H3K27me3 between 3 and 7 DAG, peaked at 3 DAG and was repressed by 7 DAG (Fig. 6D). Activation of the *LAFL* transcriptional network in mixotrophic *cs* resembled *LEC1*-overexpressing plants (Mu et al. 2008), but was independent of activation of FA catabolism and glyoxylate cycle-associated genes, which was limited to *cs* (Supplementary Fig. S11A and Supplementary Data Set 24). These observations suggested that the pathways of seed maturation and seed germination are independently regulated, but all are simultaneously activated in mixotrophic *cs*. We next asked whether repression of seed development genes before 3 DAG requires post-germination PRC2 activity, or whether H3K27me3 is already established at these genes in seeds. We found that the amount of H3K27me3 increased at selected loci following seed imbibition until 2 to 3 DAG, and similar increase was also detected in dark-grown etiolated seedlings (Fig. 6E). This indicated that H3K27me3 deposition at these genes occurs after germination, independently of light or photosynthesis. To determine if the identified sets of genes may differ in the mode of PRC2 recruitment, we analysed the enrichment of VAL1/VAL2-, TRB1-, and AZF1/BPC1-target genes and known PRE motifs among the identified subsets of H3K27me3 targets (Supplementary Fig. S11, B—D). We found that VAL1/VAL2 targets are enriched among the genes marked by H3K27me3 in 3-DAG WT that can be reactivated by sucrose in *cs* (564 genes—Fig. 6A), and among the genes that gain H3K27me3 between 3 and 7 DAG in WT but are ectopically activated in *cs* (126 genes—Fig. 6B). Genes marked by H3K27me3 in both 3- and 7- DAG WT that were reactivated in *cs* (805 genes—Fig. 6B) were enriched in all, VAL1/VAL2-, TRB1-, and AZF1/BPC1-targets and the VAL1/VAL2-bound RY-motif (CATGCA). In contrast, 378 genes marked by H3K27me3 in 3-DAG but not in 7-DAG WT (i.e. genes losing H3K27me3) were enriched in TRB1 and AZF1/BPC1 targets (Supplementary Fig. S11C), and their promoters were enriched for the REF6-binding motif (CTCTGTT) (Supplementary Fig. S11D). Therefore, VAL1/VAL2 TFs may be particularly involved in the PRC2-mediated repression of genes following seed germination.

Having identified metabolic genes that gain H3K27me3 during seedling establishment, we finally asked whether PRC2-mediated repression of metabolic genes is needed to establish photoautotrophic growth. We used CRISPR/Cas9 in *cs* to knock out *ICL* (*AT3G21720*), the key gene of the glyoxylate cycle. *ICL* is a single-copy gene that becomes transcriptionally repressed (Figs. 3D and 6D) and gains a high level of H3K27me3 between 3 and 7 DAG (Fig. 3C; Supplementary Data Set 23), It is strongly upregulated in *cs* shoot—both phototrophic (log_2_ FC cs-S-P/S-P = 8.27) and mixotrophic (log_2_ FC cs-S-M/S-M = 7.19) (Fig. 6B; Supplementary Data Set 13). We hypothesized that ectopic activation of *ICL* may prevent greening and seedling establishment, in which case *cs icl* triple mutants should green regardless of growth conditions. Indeed, several independent alleles of *cs icl* showed a significantly reduced penetrance of the TAG-accumulating phenotype and a higher frequency of transition to the vegetative phase (Fig. 6F and Supplementary Fig. S11, E—I). Therefore, PRC2-mediated transcriptional repression of *ICL* is required to promote the seed-to-seedling transition and prevent embryonic reversal in *cs*.

## Discussion

Seedling establishment involves an important developmental and metabolic transition. It is promoted by PRC2, which is evidenced by delayed seed germination (Supplementary Fig. S6D) (Bouyer et al. 2011; Müller et al. 2012) and a developmental reversal to an embryo-like state in PRC2-depleted plants (Fig. 4A and Fig. 5 and Supplementary Fig. S6, E and F) (Chanvivattana et al. 2004; Aichinger et al. 2009; Bouyer et al. 2011; Ikeuchi et al. 2015; Mozgová et al. 2017). However, how PRC2 reprograms the gene regulatory networks to prevent the developmental reversal and promote seedling establishment remains unknown. Here, we show that photosynthetic carbon assimilation is initiated between 3 to 5 DAG initiation (Fig. 1A). This coincides with the estimated depletion of storage reserves in Arabidopsis at 4 to 5 DAG (Kircher and Schopfer 2012; Xiong et al. 2013; Henninger et al. 2022) and supports a switch to photoautotrophy around the same time. In a recent study, first changes in H3K27me3 distribution in emerging seedling were observed only after 48 to 72 h after imbibition (Pan et al. 2023), which coincides with the onset of transcription of genes encoding PRC2 subunits (Mozgová et al. 2017; Pan et al. 2023). This timepoint seems to represent an important milestone in seedling establishment. Since reprogramming of H3K27me3 had not been studied beyond 72 h after imbibition (Pan et al. 2023), we focused on the transcriptome and H3K27me3 distribution in seedlings at 3 DAG (still heterotrophic) and 7 DAG (photoautotrophic) plants, identifying 2 phases of PRC2-mediated gene repression (Fig. 6G). First, before the onset of photosynthetic CO_2_ assimilation (before / by 3 DAG), H3K27me3 is deposited and ensures irreversible repression of genes transcribed during seed development and embryo maturation, including the *LAFL* TF network and storage reserve biosynthesis genes. Second, between 3 and 7 DAG, metabolic pathways related to germination and early seedling establishment are repressed.

Different TFs may target PRC2 recruitment to specific groups of genes during seedling establishment. PRC2 is recruited to cis-elements in the promoters of target genes by interacting with TFs (Xiao et al. 2017), including VAL1 and VAL2 (Yuan et al. 2021), TRB1 (Zhou et al. 2018), or AZF1 and BPC (Xiao et al. 2017). We show that H3K27me3 targets induced by external sucrose in *cs* (564 genes—Fig. 6A) and genes that gain H3K27me3 in photoautotrophic *cs* (126 genes—Fig. 6B) are exclusively enriched for VAL1/VAL2-targets (Yuan et al. 2021) (Supplementary Fig. S11B—D). Accordingly, the VAL1/VAL2-recognised RY-element is enriched among genes the gain H3K27me3 between 3 and 7 DAG. In contrast, TRB1 (Zhou et al. 2018) and AZF1/BPC1 (Xiao et al. 2017) targets and genes containing the REF6-binding motif (Pan et al. 2023) are enriched among genes losing H3K27me3 between 3 and 7-DAG (378 genes) (Supplementary Fig. S11, B—D). Mutation of REF6 did not affect H3K27me3 removal before 72 h after imbibition (Pan et al. 2023). Nevertheless, the presence of REF6-binding motifs in genes that lose H3K27me3 between 3 to 7-DAG indicates a possible contribution of REF6 to the removal of H3K27me3 after 3 DAG, which requires further experimental confirmation. Interestingly, the phenotypes of sugar-grown *clf swn* resemble *VAL* mutants *val1 val2* (*val1/2*) (Yang et al. 2013, Yuan et al. 2021) or PRC1 mutants *Atring1a Atring1b* (*Atring1a/*b) (Chen et al. 2010) and *Atbmi1a Atbmi1b Atbmi1c* (*Atbmi1a/b/c*) (Bratzel et al. 2010; Yang et al. 2013). Similarly to *cs*, these mutants accumulate TAGs and activate the *LAFL* genes in mixotrophy (Suzuki et al. 2007; Tsukagoshi et al. 2007; Bratzel et al. 2010; Chen et al. 2010; Yang et al. 2013; Yuan et al. 2021). In contrast, no TAG-accumulating phenotype has been reported in *trb1/2/3* combined mutants (Zhou et al. 2018; Wang et al. 2023) despite transcriptome similarity between *trb1/2/3* and *clf swn* (Zhou et al. 2018) and enrichment of TRB1 targets among H3K27me3-marked genes activated in photoautotrophic *cs* (805genes, Supplementary Fig. S11C). These results suggest that developmentally separated modes of PRC2 recruitment may exist during seed development and seedling establishment. VAL1/VAL2-recruited PRC2 seems particularly important for preventing the developmental reversal to embryonic state and for establishing vegetative seedling. In contrast, TRB1 and AZF1/BPC1 may facilitate establishment of H3K27me3 prior to seed germination at gene targets where H3K27me3 is retained or lost during seedling establishment. Interestingly, VAL1/2 and AtBMI1 s are required for H3K27me3 at *LAFL* genes (Yang et al. 2013) that gain the mark during seedling establishment (Fig. 6A) but not at STM or *AG* (Yang et al. 2013) that retain H3K27me3 marking throughout seedling establishment (Fig. 6B). Therefore, the different modes of PRC2 recruitment are also likely to differentially rely on PRC1. The developmentally separated or target-specific modes of PRC2 repression need to be experimentally addressed in the future.

We show that the previously reported reactivation of the embryo maturation program in PRC2-depleted seedlings (Chanvivattana et al. 2004; Aichinger et al. 2009; Bouyer et al. 2011; Yang et al. 2013; Ikeuchi et al. 2015; Mozgová et al. 2017) is conditioned by external sucrose. In the absence of sucrose, or its addition after 3 DAG, *LAFLs* transcription is stably repressed in *cs*, and no TAGs accumulate even without PRC2 (Supplementary Fig. S6, G—H). Thus, the initial downregulation of the *LAFLs* and their downstream targets is independent of PRC2, but H3K27me3 deposition is required to prevent their reactivation in a developmental time window, at which these genes can be reactivated by external stimuli (e.g. sucrose). At the *LAFL* genes, the deposition of H3K27me3 depends on VAL1/2 or BMI1A/B/C, but H2Aub does not depend on CLF/SWN (Yang et al. 2013). *val1/2* or *bmi1a/b/c* mutants retain aspects of embryo development, while *clf swn* (Yang et al. 2013) or *fie* (Bouyer et al. 2011) tend to revert to it. Therefore, VAL1/VAL2 and PRC1 may initiate the repression at *LAFLs*, while PRC2 may serve to “lock” the repressed state. This mechanism is reminiscent of the repression of *FLC*, where VAL1-mediated recruitment of a HDAC and PRC1 promote the recruitment of PRC2 (Questa et al. 2016; Mikulski et al. 2022) and stabilization of *FLC* repression (Whittaker and Dean 2017). HDAC and PRC activities can be recruited using a synthetic VAL1- but not PRC-recruiting transgene (Baile et al. 2021), supporting the leading role of VAL1 in gene repression. Whether VAL1/VAL2 initiate the repression of *LAFLs* during seedling establishment and how the reactivation of *LAFLs* is prevented by initial absence of exogenous sucrose remain to be established.

In addition to promoting the developmental transition from seed to seedling, the timing of H3K27me3 deposition during seedling development may limit the potential of developmental reprogramming. Indeed, we found that within the first 7 days, H3K27me3 represses a number of *TFs* instructive for embryo, root or shoot development and regeneration (Figs. 2A and 6, A and B, Supplementary Data Sets S7 and S22 and S23). Beside *LEC1* (Lotan et al. 1998) and *LEC2* (Stone et al. 2001), these include for example *WUS* (Zuo et al. 2002; Zhang et al. 2017), *CUC1/2* (Daimon et al. 2003), *PLT4/BBM* (Boutilier et al. 2002; Horstman et al. 2017), *AGL15* (Thakare et al. 2008), *PLT3*, *PLT5/EMK*, and *PLT7* (Tsuwamoto et al. 2010; Kareem et al. 2015). In agreement with H3K27me3 deposition at *LAFLs* before 3 DAG, the potential to induce somatic embryo development, associated with *LAFL* reactivation (Jia et al. 2013; Horstman et al. 2017), is often restricted to seed germination or early seedling development (Horstman et al. 2017; Mozgová et al. 2017). Deposition of H3K27me3 during early seedling establishment may therefore limit the time window of regenerative potential in plant tissues.

The deposition of H3K27me3 before 3 DAG is independent of photosynthesis and light signaling (Fig. 6E), and is mostly insensitive to the photosynthetic inhibitor DCMU (Fig. 2A). The 3-DAG seedlings were not yet assimilating photosynthetic sugar (Fig. 1A), suggesting that they were photosynthetically inactive and therefore the impact of DCMU was limited. In contrast, the levels of H3K27me3 dramatically decreased in the shoot and root of heterotrophic 7-DAG plants (Fig. 2A and Supplementary Fig. S3, A and B). In this system, exogenous sucrose and DCMU were provided to light-grown plants to force heterotrophy. DCMU, that blocks electron transport from PSII to PQ_B_, also induces ROS production (Flors et al. 2006). Accordingly, we found that oxidative stress responses are activated in the DCMU-grown plants (Supplementary Data Sets S2 and S3). Therefore, it is possible that the observed decrease of H3K27me3 and transcriptional response in 7-DAG heterotrophic plants reflects a general oxidative stress response, rather than heterotrophic mode of nutrition and growth. Indeed, the distribution of H3K27me3 or transcriptome of 7-DAG DCMU-grown (heterotrophic) shoot (S-H) was distinct from the 3-DAG shoot (S-P3 or S-D3) (Supplementary Fig. S1, A and B), further corroborating this notion. We did not observe changes in the transcription of PRC2 subunits or H3K27me3 demethylases that would be consistent in the shoot and the root and could directly explain the comparable effect of DCMU on global H3K27me3 levels in both the tissues (Supplementary Fig. S3C). Thus, the mechanisms that led to H3K27me3 depletion in DCMU-treated sucrose-grown plants requires further investigation. Interestingly, transcriptional changes indicating elevated oxidative stress in photoautotrophic *cs* shoots resembled those observed in the shoot of DCMU-grown 7-DAG plants (Fig. 4I). Metabolic and transcriptomic changes in *cs* (Figs. 4 and 5) are also reminiscent of changes that occur in plants with reduced activity of TOR (Caldana et al. 2013). Strong inhibition of TOR inhibits nuclear PRC2 activity (Ye et al. 2022) and reduces global levels of H3K27me3 (Ye et al. 2022; Dong et al. 2023). It will be important to determine whether TOR signaling contributes to the DCMU-induced H3K27me3 depletion and how H3K27me3 depletion in *cs* feeds back into the potential crosstalk.

Primary metabolic genes are targeted by PRC2 during the transition to vegetative growth. These include important genes involved in lipid metabolism, the glyoxylate cycle or gluconeogenesis in the shoot, as well as photosynthesis-related genes in the root (Fig. 3). Several observations in this work indicate that developmental identity can be underpinned by metabolic state. First, we found that inhibition of photosynthesis in the shoot is associated with the upregulation of photosynthesis-related genes and downregulation of root development in the root (Supplementary Data Set 3). Second, the development of TAG-filled cotyledon-like structures in *cs* plants, instead of photosynthetically active true leaves, is promoted by the addition of exogenous sucrose (Fig. 4A and Supplementary Fig. S6, E—H). Third, preventing the transcription of *ICL* can promote vegetative transition in *cs* plants (Fig. 6F). Although the molecular mechanisms are not yet clear, these results demonstrate a close link between the developmental and metabolic identities controlled by PRC2.

## Materials and methods

### Plant material and cultivation conditions

Arabidopsis (*A. thaliana*) wild-type (WT) (Col-0) plants and strong PRC2 mutant alleles *clf-29* (SALK_021003) (Bouveret et al. 2006) and *swn-3* (SALK_050195) (Chanvivattana et al. 2004) were used in most experiments. For confirmation other strong PRC2 mutant alleles *clf-28 swn-7* (*clf-28:* SALK_139371; *swn-7*: SALK_109121) (Lafos et al. 2011; Gan et al. 2015), and *fie cdka;1* (*fie*: SALK_042962; *cdka;1*: SALK_106809) (Bouyer et al. 2011) were used. The combination of the used *clf* and *swn* alleles induce a severe developmental phenotype (Lafos et al. 2011; Mozgová et al. 2017), preventing homozygous seed production. Thus, *clf swn* (*cs*) double mutant plants were selected from a segregating population of seeds produced by *CLF/clf swn/swn* (*Ccss*) parental plants. The *cs* phenotype/genotype can be faithfully distinguished at 3 DAG induction. Parental generations of compared plants were grown concurrently under the same growth conditions.

The following cultivation conditions were used unless specified otherwise. The seeds were surface sterilized by ethanol and placed on one of the following media: “P” (photoautrophic): ½ MS (Murashige & Skoog medium: ½ Murashige & Skoog (MS) medium including vitamins (Duchefa cat.no. M0222), 2-(N-morpholino)ethanesulfonic acid and 0.8% (w/v) plant agar (Duchefa cat.no. P10011000)), “D” (DCMU): ½ MS + 10 *μ*M DCMU (3-(3,4-dichlorophenyl)-1,1-dimethylurea: Diuron—Sigma Aldrich cat.no. D2425), “M” (mixotrophic): ½ MS + 30 mm (1%) sucrose; “H” (hetrotrophic): ½ MS + 30 mm (1%) sucrose + 10 *μ*M DCMU. For ^13^C analyses, MS medium without vitamins was used (Duchefa cat.no. M0221). Sterile layer of cellophane was placed on the top of the solidified medium to avoid medium-origin carbon contamination. For selected experiments, 30 mm mannitol was used as osmotic control. The seeds were stratified over 2 nights (ca 65 h) and placed in long-day conditions (110 to 120 *µ*mol m^−2^ s^−1^, 16 h light/8 h dark, 22 °C during light/20 °C during dark; light source: OSRAM FQ 54W/840 HO CONSTANT LUMILUX Cool White). Germination (radicle emergence) was scored at given time points, presence of *clf swn* phenotype in the population of *CLF/clf swn/swn* progeny was scored at 10 DAG induction. Plant in soil for seed amplification and scoring seed set and abortion rate were cultivated under long-day conditions (110 *µ*mol m^−2^ s^−1^, 16 h light/8 h dark, 21 °C).

### Generation of CRISPR-ICL lines

To target *ICL* (*AT3G21720*), 2 suitable sgRNA targeting *ICL*—*exon 2* were designed using CRISPOR (Concordet and Haeussler 2018) and cloned in BpiI sites of pDGE Shuttle vectors (Addgene plasmid #153241 pDGE332 and Addgene plasmid #153243 pDGE334) before assembly into BsaI sites of pDGE347 (Addgene plasmid #153228) using the method previously described (Stuttmann et al. 2021). All sgRNAs and oligonucleotides are listed in (Supplementary Data Set 25).The binary vector was transferred to *Agrobacterium tumefaciens* (GV3101) for transformation by floral dipping (Clough and Bent 1998) of *CLF/clf-29 swn-3/swn-3* (*Ccss*) plants. RFP(Cas9)-positive T1 seeds were selected using Leica S9i (Leica, DE) stereomicroscope with NIGHTSEA fluorescence adaptor (Nightsea, USA) and cultivated in soil. *Ccss* plants were selected and *ICL* locus containing the targeted sites was sequenced by Sanger sequencing (Supplementary Data Set 25). RFP(Cas9)-negative T2 seeds were selected and *Ccss icl* (homozygous) mutant plants were selected. T3 generation *ccss icl s*eedlings of 10 independent lines were analysed that showed comparable phenotypes, 4 lines are shown.

### Detection of TAGs using Sudan red 7B (fat red)

21-DAG plants were analysed for the presence of embryonic lipids using Sudan Red 7B (Sigma-Aldrich, #46290) as described by Aichinger et al. (2009). Briefly, seedlings were immersed in filtered solution of 0.5% (w/v) Sudan Red 7B in 60% isopropanol for 15 min and rinsed 3 times with H_2_O. The penetrance of fat-red phenotype was calculated as percentage of red-staining plants. Phenotypes were documented using Optika stereo-microscope equipped with optikam PRO8 Digital Camera (Optica, IT) or Leica S9i stereomicroscope (Leica, DE).

### Determination of CO_2_ assimilation

Two approaches were taken to assess CO_2_ assimilation. To assess the onset of CO_2_ assimilation (Fig. 1A), we used natural ^13^C fractionation, exploiting the different isotopic composition of CO_2_ in atmospheric air (δ^13^C = −8.5‰) and artificial air (δ^13^C = −40‰). SSs of plants grown in atmospheric air (δ^13^C seeds −30‰, reflecting metabolic ^13^C discrimination (Cernusak et al. 2013)) were placed into a desiccator continually flushed with artificial air (mixture of ∼80% N_2_, 20% O_2_ and 0.04% CO_2_; δ^13^C = −40‰) and plants were grown for up to 14 DAG. Decrease in δ^13^C in 14 DAG plants results in δ^13^C_shoot_ —55‰ and δ^13^C_root_ —50‰, reflecting the ^13^C signal of assimilated CO_2_ in artificial air, ^13^C discrimination during photosynthesis (Farquhar et al. 1989) and post-photosynthetic discrimination (Cernusak et al. 2009). To assess the level of CO_2_ assimilation in 1 h (Fig. 4B), a ^13^C-labeling approach was carried out as previously described by Kubásek et al. (2021). In particular, seedlings were cultivated under standard long-day conditions (as specified above) for 10-days and ^13^C labeling was carried out at ZT 7 to 8 (middle of photoperiod). Open plates with seedlings were placed in a 3-L chamber made of an inverted glass petri dish with water at the bottom to seal the gap between the dish and the lid against gas leakage. The chamber was equipped with a sealed metal tube to allow gas injection and a ventilator for homogeneous gas distribution. The chamber was flushed with 5 L of CO_2_-free air (80% nitrogen, 20% oxygen) to remove ambient CO_2_. Next, 3 mL of ^13^CO_2_ (99% ^13^C, 0% ^12^C) were injected into the chamber (final CO_2_ concentration was 1000 *µ*mol.mol^−1^), after which the plants were incubated in the closed chamber for 1 h (pulse). For both approaches, shoots and roots were dissected and air-dried to obtain final 0.1 to 0.2 mg DW material per sample. Samples were combusted in oxygen (elemental analyser Flash 2000, Thermo Scientific, Brehmen, Germany) and the ^13^C/^12^C ratio in the resultant CO_2_ was determined by isotope ratio mass spectrometer (Deltaplus XL, Thermo Scientific, Brehmen, Germany). Each experiment was performed in biological triplicates.

### Biomass analysis

WT (Col-0) and *clf-29 swn-3* seeds were sterilized and plated on ½ MS medium plates “P” (photoautrophic) and “M” (mixotrophic) and cultivated under standard long-day conditions (as previously described). For the biomass analysis WT and *cs* seedling were sampled at 6 DAG and 12 DAG. The fresh weight of the seedlings was assessed for the biomass analysis. The experiment was performed using 4 biological replicates.

### ABA concentration measurements

Extraction and purification of ABA were done using a previously described method (Ture”ková et al. 2024) with minor modifications. Frozen samples (5 mg fresh weight) were homogenized using a MixerMill (Retsch GmbH, Haan, Germany) and extracted in 1 mL of ice-cold extraction solvent methanol/water/acetic acid (10/89/1, v/v/v) and stable isotope-labelled internal standard (5 pmol of ^6^H_2_-ABA per sample added). The extracts were purified on Oasis HLB columns (30 mg, Waters Corp., Milford, USA), conditioned with 1 mL methanol and equilibrated with 1 mL methanol/water/acetic acid (10/89/1, v/v/v). After sample application, the column was washed with 1-mL methanol/water/acetic acid (10/89/1, v/v/v) and then eluted with 2 mL methanol/water/acetic acid (80/19/1, v/v/v). Eluates were evaporated to dryness and dissolved in 30 μL of mobile phase prior to mass analysis using an Acquity UPLC System and triple quadrupole mass spectrometer Xevo TQ MS (Waters, Milford, MA, USA) (Floková et al. 2014).

### Plant material for transcriptome, H3K27me3 and metabolome profiling

WT (Col-0) and *clf-29 swn-3* (*cs*) were used for all profiling experiments. Plants for profiling were cultivated within the same period of time and material harvested at the same time was divided to be used in all profiling experiments. Biological replicates were cultivated on separate plates, that were regularly moved within the cultivation chamber to randomize the environmental effects. For RNA-seq and ChIP-seq, WT plants were harvested at 3 and 7 DAG. Due to 2- to 3-day delay in the germination, *cs* plants were harvested at 9-DAG (growth shifted forward, harvesting done at the same time as 7-DAG WT). Separated shoots and roots were collected by seedling dissection, and the same seedling pool was used as the corresponding shoot and root sample for RNA-seq and ChIP-seq. In 3-DAG samples, material from 200 or 400 seedlings per replicate was pooled for RNA- or ChIP-seq, respectively (material from 400 seedlings corresponded to ca 50 mg FW for either tissue). In 7-DAG WT, material from 100 or 200 seedlings per replicate was pooled for RNA- or ChIP-seq, respectively, and 50 mg FW was used in ChIP. In 9-DAG *cs* samples, material from 150 to 200 seedlings per replicate was pooled for RNA extraction. Replicates A, B, and C were harvested at zeitgeber time (ZT) 4 to 7, ZT 7 to 10 and ZT 10 to 13, respectively. Each individual sample was collected into RNAlater (Thermo, ct. no. AM7020) and flash frozen in liquid nitrogen for RNA extraction or immediately crosslinked and flash frozen for ChIP. All replicates were used for RNA-seq and but only replicates B and C used in ChIP-seq, while replicate A was retained for qPCR confirmation. For primary metabolome profiling, 5 biological replicates of 20 mg FW shoot tissue per replicate were dissected from 7-DAG WT and 9-DAG *cs* seedlings. Collection was performed at ZT 4 to 6. Following shoot collection, the tissue was washed by MilliQ and dried on filter paper before flash freezing in liquid nitrogen.

Seed stages for metabolome profiling were harvested from the main inflorescence of WT (Col-0) plants grown on soil. Siliques were tagged at pollination (representing stage 1) and harvested in pools of 2 to 3 successive siliques per stage, obtaining 25 stages along the main inflorescence stem (stage 25 representing the most mature siliques in the bottom part of the inflorescence stem). In stages 6 to 25, developing seeds were dissected from the lyophilized siliques. Earlier stages were combinations of siliques and developing seeds. Samples were pools of 3 mg FW, representing a pool of 170 to 200 plants each. Three independent replicates were harvested at each day after pollination, day 22 had 2 replicates, days 24 and 25 yielded only a single replicate.

### Gene transcription quantification by RT-qPCR

WT (Col-0) and *clf-29 swn-3* seeds were sterilized and plated on ½ MS medium plates “P” (photoautrophic) and “M” (mixotrophic) and cultivated under standard long-day growth conditions (as previously described). WT seedlings were harvested at 2 DAG (∼200 seedlings per replicate) and 7 DAG (∼100 seedlings per replicate), and *cs* seedlings were harvested at 9 DAG (∼200 seedling per replicate) at ZT 4 to 7. Each sample was collected separately into RNAlater (Thermo, ct. no. AM7020) and immediately flash frozen in liquid nitrogen for RNA extraction. 50 mg of collected material from each sample was used for the RNA extraction. RNA was extracted using MagMAX Plant RNA Isolation Kit (Thermo, cat.no. A33784). 1 *μ*g of extracted RNA was reverse-transcribed using the RevertAid First Strand cDNA Synthesis Kit (Thermo, cat.no. K1622) with Oligo (dT)_18_ primers. RT-qPCR was performed using the CFX Connect Optics Module Real Time PCR detection system (BIO-RAD) with gene-specific primers (Supplementary Data Set 25) and 5× HOT FIREPol Eva Green qPCR Mix Plus (ROX) (Solis Biodyne, cat.no. 08–25-00008). The experiment was performed using 3 biological replicates. *PP2A* (*AT1G13320*) was used as reference gene. The 2^−ΔΔCt^ method (Livak and Schmittgen 2001) was used to quantify relative transcript abundance. R programming language version R 4.3.1 (R Core Team 2023) was used for statistical computing and graphics. Outliers were excluding using Dixon's (*p*-value threshold = 0.05) tests with the R package Outliers (Komsta 2022 ). Two-way ANOVA tests were performed with base R and Tukey's HSD tests were performed with emmeans (Lenth 2016) and mulcomp (Hothorn et al. 2008) and heatmaps were produced with pheatmap (Kolde 2019), RColorBrewer (Neuwirth 2022), ggplotify (Yu 2019 ), and ggplot2 (Hadley 2016) R packages. Hierarchical clustering was based on row *Z*-score-normalized transcript abundance of genes in 2- and 7-DAG photoautotrophic (S-P2 and S-P) and mixotrophic (S-M2 and S-M) WT shoot and 9-DAG photoautotrophic (*cs*-S-P) and mixotrophic (*cs*-S-M) *cs* shoot.

### RNA-Seq library preparation, sequencing and data analyses

Total RNA was extracted from root and shoot tissues using MagMAX Plant RNA Isolation Kit (Thermo, cat.no. A33784). polyA-mRNA was enriched using NEBNext Poly(A) mRNA Magnetic Isolation Module (NEB, cat. No. E7490) and Illumina sequencing libraries were prepared using NEBNext Ultra II RNA Library Prep Kit for Illumina (NEB, cat. No. E7770), 24 libraries were pooled and sequenced on Illumina HiSeq4000 in 50 bp single-end (SE) mode. Obtained numbers of reads per sample are available in (Supplementary Data Set 1). Raw RNA-seq data were trimmed using TrimGalore! v0.4.1 (https://github.com/FelixKrueger/TrimGalore, v0.4.0), quality checked using FastQC v0.11.5 (Andrews et al. 2010) and mapped to *A. thaliana* TAIR10 genome using Hisat2 v2.0.5 (Kim et al. 2019). Gene expression was quantified as RPKM (reads per kilobase of the gene per million of the reads in the dataset) in Seqmonk v1.40.0 (https://www.bioinformatics.babraham.ac.uk/projects/) using Araport11 annotation. Differential gene expression was analysed using DESeq2 and EdgeR within Seqmonk v1.40.0 with *P*-value cut-off 0.05. Genes were considered significantly differentially expressed if they were identified as such by both DESeq2 and EdgeR and the expression fold change was at least ± 1.5 (abs. log_2_FC 0.6).

### Chromatin immunoprecipitation and qPCR

Chromatin immunoprecipitation was carried out using previously described protocol (Mozgová et al. 2015). In brief plant material was crosslinked using 1% formaldehyde for 10 min under vacuum and crosslinking was terminated using 0.125 m glycine for 5 min. Sheared chromatin (10 cycles, 30 s ON/ 30 s OFF using a Bioruptor (Diagenode)) extracted from 100 mg material was equally divided between input and samples immunoprecipitated with antihistone H3 (Merck, cat. no. 07-690); anti-H3K27me3 (Merck, cat. no. 07-449) and IgG (Merck, cat. no. I5006) as control. Immunocomplexes were collected using Dynabeads Protein A for Immunoprecipitation (Thermo, cat. no. 10,001D) and washed 2×5 min with low-salt buffer (containing 150 mm NaCl), 2×5 min with high-salt buffer (containing 500 mm NaCl) and 1×5 min with LiCl-containing buffer. Recovered DNA was extracted using phenol-chloroform and precipitated by ethanol with addition of GlycoBlue Coprecipitant (Thermo, cat. no. AM9515) and purified DNA was analyzed by qPCR using CFX Connect Real-Time PCR Detection System (Bio-Rad) using 5X HOT FIREPol Eva Green qPCR Mix Plus (ROX) (Solis Biodyne) with gene-specific primers detailed in (Supplementary Data Set 25). ChIP performance was quantified by comparing immunoprecipitated DNA to input and abundance of H3K27me3 is shown as anti-H3K27me3 or IgG related to anti-H3 recoveries (H3K27me3/H3 or IgG/H3).

### ChIP-seq library preparation, sequencing and data analyses

For ChIP-seq, immunoprecipitated DNA was purified using iPure kit v2 (Diagenode, cat. no. C03010015). Following qPCR control to assess locus-specific signal/noise ratio, DNA amount in input, anti-H3 and anti-H3K27me3 immunoprecipitated was quantified using Qubit dsDNA HS Assay (Thermo, cat. no. Q32854) and 3 ng of DNA was used for Illumina sequencing library preparation using NEBNext Ultra II DNA Library Prep Kit for Illumina NEB, cat. no. E7645S) according to manufacturer’s instructions. Libraries were pooled into 2 pools of 24 samples (expected output min 20 million reads per sample) and sequenced on the Illumina HiSeq Hi-Outputv4 platform in 125 bp paired-end (PE) mode. Obtained numbers of reads per sample are available in (Supplementary Data Set 4). To assess the quality of the sequence (raw and after trimming), FastQC v0.11.5 (Andrews et al. 2010) was used. To clean the raw data, TrimGalore v0.6.2 was used, with parameters: Paired; Trim N; Phred33; Stringency 6; Quality 20; Minimum length 20; Clip 10 bp from 3′ and 5 bp from 5′. Raw ChIp-Seq reads were mapped to TAIR10 *A. thaliana* genome using Bowtie2 v2.2.9 enabling the option –no-mixed. The SAM file was processed (mapping quality filter, with a MAPQ threshold of 25, conversion to BAM format, sorting and indexing) using SAM tools v1.9. Coverage of input, H3 and H3K27me3 was computed using deepTools2—BAMcoverage (Ramírez et al. 2016). Reads were extended to 200 bp (estimated mean library insert size), normalized by BPM (bins per million mapped reads), bin size was 50 bp. Enrichment of H3K27me3/H3 was computed per 50 bp bin (in log_2_ ratio) with deepTools2—BAMcompare taking H3K27me3 as sample and H3 as control (Ramírez et al. 2016). Average enrichment per feature (annotated gene) was calculated using DeepTools2—multiBigwigSummary (Ramírez et al. 2016), using H3K27me3/H3 enrichment values. BedTools v2.26.0 was used to parse the BAM format to BED format. SICER (Xu et al. 2014) was used to identify enriched regions (peaks) for H3K27me3, broad modification, with the following parameters: Redundancy threshold 25; Window size 200; Gap 600; Effective genome size 0.998444; FDR 0.05; Fragment size 150; Control: H3 enriched sample; Sample: H3K27 enriched sample. To relate features (genes) to identified enrichment peaks, BedTools v2.26.0 (Intersect) and Araport11 annotation were used (Cheng et al. 2017). To identify a feature as covered by a peak, at least 1 bp of the feature must be covered by the peak (T1 genes), or ≥70% of feature body must be covered by the peak (T70 genes). A peak could cover more than 1 feature. PCA and Sperman’s correlation plots were produced using average BPM over gene bodies of all H3K27me3-marked genes (T70 genes) found in at least 1 of the samples, using DeepTools3.5.1 (plotCorrelation and plotPCA). Results of ChIP-seq peak-calling analyses are available through BioStudies (https://www.ebi.ac.uk/biostudies/) under the accession S-BSST1976 (doi:10.6019/S-BSST1976).

### Gene ontology enrichment and TF gene identification

Gene ontology enrichment analysis was performed using DAVID 6.8 (Langmead and Salzberg 2012). GO categories passing the thresholds of Bonferroni-corrected *P*-value ≤ 0.05 and min. enrichment ≥ 1.5 were considered. Bubble plots of GO-enriched pathways were plotted using SRplot (Tang et al. 2023). TF-gene association was based on Plant Transcription Factor Database (PlantTFDB—https://planttfdb.gao-lab.org/) and TF genes defined by Czechowski et al. (2004).

### Enrichment of VAL1/VAL2, TRB1 and AZF1/BPC1 targets, and PRE motif enrichment

Lists of gene targets of VAL1/VAL2 (Yuan et al. 2021) and TRB1 (Zhou et al. 2018) were taken from the respective studies. AZF1 and/or BPC1 targets were determined from the center of ChIP-peak coordinates (Xiao et al. 2017) using ChIPSeeker R package (Wang et al. 2022). Peaks centered within 3 kb upstream and 250 bp downstream of TSS of Araport11-annotated gene were assigned to that gene. In case of multiple assignments, the gene whose TSS was the closest was retained. 9,036 genes marked by H3K27me3 in 3- and/or 7-DAG shoot of WT seedlings were used as background. PRE and REF6-binding motif enrichment was analysed in promoter sequences (−1,000 to 0 bp from TSS), retrieved by RSAT (TAIR10 assembly) (Nguyen et al. 2018). Position weight matrices were obtained from publications by Zhou et al. (2018) and Yuan et al. (2021). Promoter sequences of the whole genome were used as a background and the *P*-values were computed with the sea (simple enrichment analysis) command of the meme suite (Bailey et al. 2015), considering a 3-order model (options: –order 3 –align right –hofract 0.3).

### Metabolite extraction and data analysis

Deep frozen plant shoot samples of ∼20 mg fresh weight (FW) or ∼3 mg (FW) of developing seed material were homogenized in the frozen state by an oscillating ball mill, extracted by precooled solvents, chemically derivatized, and analyzed by gas chromatography—electron impact ionization—mass spectrometry (GC-MS) as described previously by Erban et al. (2020). In detail, 160 *µ*L polar phase of an extraction mixture of 360:400:200 (v/v/v) methanol:water:chloroform was obtained after thorough mixing with the frozen powder and separation of liquid phases by centrifugation for 5 min at 20,800 × *g*. ^13^C_6_-Sorbitol was added as internal standard to the extraction mixture. The 160 *µ*L aliquot was dried in a vacuum concentrator and stored at −20 °C until further processing. In the case of seedling analysis, the complete polar phase was sampled and dried. Before GC-MS injection, dry samples were methoxyaminated by methoxyamine hydrochloride dissolved in pyridine to a final concentration of 40 mg × mL^−1^ and subsequently trimethylsilylated by N,O-bis(trimethylsilyl)trifluoroacetamide (Erban et al. 2020). A standard mixture of *n*-alkanes, *n*-decane (RI 1000), *n*-dodecane (RI 1200), *n*-pentadecane (RI 1500), *n*-octadecane (RI 1800), *n*-nonadecane (RI 1900), *n*-docosane (RI 2200), *n*-octacosane (RI 2800), *n*-dotriacontane (RI 3200), and *n*-hexatriacontane (RI 3600), was prepared in pyridine and added to each derivatized sample for retention index calibration. Metabolite profiles were performed by an Agilent 6890N gas chromatograph with split/splitless injection and electronic pressure control (Agilent, Böblingen, Germany) using a 5% phenyl–95% dimethylpolysiloxane fused silica capillary column of 30 m length, 0.25 mm inner diameter, 0.25 *μ*m film thickness, an integrated 10 m precolumn and helium carrier gas for chromatographic separation. Electron impact ionization and time-of-flight MS were performed with a Pegasus III time-of-flight mass spectrometer (LECO Instrumente GmbH, Mönchengladbach, Germany) (Erban et al. 2020).

ChromaTOF software (version 4.22; LECO, St. Joseph, USA) and TagFinder software (Luedemann et al. 2008) were used for chromatogram processing and compound annotation. Analytes, i.e. chemically derivatized metabolites, were annotated manually using TagFinder software by matching of mass spectral and chromatographic retention index information to reference spectra and retention indices of authenticated reference compound from the Golm Metabolome Database (http://gmd.mpimp-golm.mpg.de), metabolite, analyte, and match quality information are reported (Supplementary Data Sets S17 and S18). Each sample was analyzed in both, splitless and split (1:30 ratio) mode. Abundant compounds that were above the upper limit of quantification in splitless mode were re-analyzed by split mode. Acquired metabolite abundances of arbitrary units were background-subtracted by nonsample control measurements, normalized to the abundance of the internal standard ^13^C_6_-sorbitol and to the sample FW of each sample for relative quantification by normalized metabolite responses, arbitrary units g^−1^ (FW).

R programming language version R 4.3.1 (R Core Team 2023) was used for statistical computing and graphics. Outliers were excluding using Rosner's generalized extreme studentized deviate procedure (maximum number of outliers = 2, *P*-value threshold = 0.05) with PMMCRplus (Pohlert 2023). Two-way ANOVA with Tukey's HSD test and heatmaps were produced using R packages as described in previous section. Hierarchical clustering is based on row *Z*-score-normalized relative abundances of 85 metabolites in WT photoautotrophic (S-P) and mixotrophic (S-M) shoot and *cs* photoautotrophic (cs-S-P) and mixotrophic (cs-S-M) shoot samples. PCA of metabolite profiles (Supplementary Data Set 17) was performed in R 4.1.1 (R Core Team 2021). Normalized response data were log_10_-transformed, mean-centered and auto-scaled prior to PCA and processed using the packages ggplot2 (Hadley 2016), ggpubr (Kassambara 2020), and factoextra (Kassambara and Mundt 2020).

WT and *cs* mutant profiles were compared with time series of *A. thaliana* seed maturation (Supplementary Data Set 18) and germination data (Ginsawaeng et al. 2021) by Spearman rank correlation across the shared metabolites among the data sets (Supplementary Data Set 21). Metabolite data were maximum-scaled per metabolite and dataset to a range of 0 to 100. All data were from GC-MS profiling analyses except amino acid abundances of the germination series that were LC-MS measurements (Ginsawaeng et al. 2021). Correlation coefficients of all sample combinations were arrayed in a symmetric matrix, tested for the significance of correlation (*P* > 0.05) and visualized as a 3-color heat map in the range of negatively correlated (−1) in blue, noncorrelated (0) in white, and positively correlated (+1) in red. Matrices of Spearman rank correlation coefficients from primary metabolome profiles of sucrose-supplemented WT, mannitol-supplemented mutant and sucrose-supplemented mutant correlated to the profiles of seed-maturation and seed-germination stages differed from mannitol-supplemented WT in a characteristic developmental stage dependent manner (Supplementary Data Set 21). These differences of correlation coefficient matrices were tested at each developmental stage by the heteroscedastic Student’s *t*-test of the MS-EXCEL table calculation program and confirmed by Wilcoxon rank sum testing using the R function wilcox.test of the stats package (R Core Team 2021). The correlation coefficient matrices were generated from correlation calculations of all available replicate measurements, namely 3 to 5 replicate profiles per seed maturation stage from this study or 5 replicates of the seed germination stages (Ginsawaeng et al. 2021) correlated to 6 replicates of the WT and mutant seedlings of this study, respectively. With few exceptions, normality of the correlation coefficient matrices, treatment groups versus developmental stages, was confirmed by Shapiro-Wilk testing, as well as heteroscedasticity by the Levene's test (*P* ≤ 0.05).

Modules (=clusters) and metamodules (=metaclusters) of metabolites (Supplementary Fig. S10) were obtained using mean values maximum-scaled per metabolite and dataset. Weighted metabolites correlation network analysis implemented with the WGCNA R package (Langfelder and Horvath 2008) was performed. Weighted networks rely on metabolite adjacencies, which correspond to absolute metabolite correlations exponents of a soft-thresholding power *β*. The value *β* = 7 was chosen to obtain a network with Scale-Free Topology properties (Scale Free Toplogy Model Fit *R*^2^ ≥ 0.6) and high connectivity. Adjacency values were used to compute the Topological Overlap Matrix (TOM). Metabolites were clustered into modules based on the (1-TOM) distance matrix, by hierarchical clustering with average linkage distance comparison between clusters. The number of modules was determined by Dynamic Hybrid tree cut (dendrogram cut height for module merging = 0.25). Metamodules were identified based on the results of hierarchical clustering of the eigenmetabolites representing each module, considering the (1-TOM) distance.

### Statistical analyses and plotting

Statistical analysis and graph generation were performed using OriginPro 2023, version 10.0.0.154 (Academic) and R version R 4.1.1 (R Core Team 2021) and version R 4.3.1 (R Core Team 2023). Source data used for graph generation depicting experimental datasets with fewer than 6 biological replicates are included in Supplementary Data Set 26.

### Accession numbers

Sequence data from this article can be found in the GenBank/EMBL data libraries under accession numbers *AT2G23380* (*CLF*)*, AT4G02020* (*SWN*)*; AT3G21720* (*ICL*) and gene accession numbers listed in Supplementary Data Sets.

## Supplementary Material

koaf148_Supplementary_Data

## Data Availability

The data for this study have been deposited in the European Nucleotide Archive (ENA) at EMBL-EBI under accession number PRJEB80531 (https://www.ebi.ac.uk/ena/browser/view/PRJEB80531).

## Supplementary data

The following materials are available in the online version of this article.

**Supplementary Figure S1.** RNA-seq and ChIP-seq technical controls. (Supplementary Supports Figure 1).

**Supplementary Figure S2.** ChIP-seq technical controls: a high level of H3K27me3 enrichment is associated with low transcription. (Supplementary Supports Figure 1).

**Supplementary Figure S3.** Coverage of H3 and H3K27me3 in H3K27me3 target genes identified in the shoot or root tissues. (Supplementary Supports Figure 2).

**Supplementary Figure S4.** Shoot- and root-specific H3K27me3 targets and the impact of photosynthesis inhibition on H3K27me3 enrichment. (Supplementary Supports Figure 2).

**Supplementary Figure S5.** ChIP-qPCR confirmation of ChIP-seq and GO of genes that lose H3K27me3 and are activated between 3 and 7 DAG. (Supplementary Supports Figure 3).

**Supplementary Figure S6.** The absence of CLF and SWN does not reduce embryo or seed viability but is associated with delayed seed germination and sucrose-dependent TAG accumulation. (Supplementary Supports Figure 4).

**Supplementary Figure S7.** Transcriptome analysis of *clf swn* cultivated under photoautotrophic or mixotrophic conditions and ABA concentration in the samples. (Supplementary Supports Figure 4).

**Supplementary Figure S8.** GO enrichment of the biological processes commonly up- or down-regulated in photoautotrophic *cs* and DCMU-grown WT. (Supplementary Supports Fig. S4H).

**Supplementary Figure S9.** Comparison of transcriptome and primary metabolome data. (Supports Fig. 5).

**Supplementary Figure S10.** WGCNA of the primary metabolome profiles from 25 seed maturation stages, dry seeds, and 5 seed germination stages. (Supports Fig. 5).

**Supplementary Figure S11.** PRC2 recruitment modules involved in the seed-to-seedling transition and CRISPR/Cas9-mediated mutagenesis of *ICL* in *cs*. (Supports Fig. 6).

**Supplementary Data Set 1.** RNA-seq read and alignment statistics. (Related to Fig. 1).

**Supplementary Data Set 2.** Analysis of DEGs in WT shoots and associated GO terms. (Related to Fig. 1C).

**Supplementary Data Set 3.** Analysis of DEGs in WT roots and associated GO terms. (Related to Fig. 1C).

**Supplementary Data Set 4.** ChIP-seq read and alignment statistics. (Related to Fig. 1, Supplementary Fig. S1).

**Supplementary Data Set 5.** Lists of H3K27me3 target genes (T1, T70) identified in this study. (Related to Fig. 1D).

**Supplementary Data Set 6.** GO analyses of H3K27me3 (T70) target genes. (Related to Fig. 1D).

**Supplementary Data Set 7.** Effect of heterotrophy (DCMU + 1% sucrose) on 7-DAG WT shoot and root H3K27me3 targets. (Related to Fig. 2B).

**Supplementary Data Set 8.** Effect heterotrophy (DCMU + 1% sucrose) on genes with changed H3K27me3 enrichment between 3- and 7-DAG shoots and roots. (Related to Fig. 2, C and D).

**Supplementary Data Set 9.** Comparison of H3K27me3 enrichment in 3- and 7-DAG WT shoots and GO terms associated with genes showing altered H3K27me3 between 3 and 7 DAG. (Related to Fig. 3A).

**Supplementary Data Set 10.** List of H3K27me3 targets (T70) that change in expression between 3- and 7-DAG shoots. (Related to Fig. 3E).

**Supplementary Data Set 11.** Comparison of H3K27me3 enrichment in 3- and 7-DAGDAG WT roots and GO terms associated with genes showing altered H3K27me3 between 3 and 7 DAG. (Related to Fig. 3F).

**Supplementary Data Set 12.** List of H3K27me3 targets (T70) that change in expression between 3- and 7-DAG roots. (Related to Fig. 3J).

**Supplementary Data Set 13.** Lists of DEGs in *clf swn* (*cs*) mutant seedling shoots and associated GO terms. (Related to Fig. 4E, Supplementary Fig. S7C).

**Supplementary Data Set 14.** Lists of DEGs in *clf swn* (*cs*) mutant seedling roots and associated GO terms. (Related to Fig. 4G, Supplementary Fig. S7D).

**Supplementary Data Set 15.** Lists of DEGs in photoautotrophic (*cs*-S-P) and mixotrophic (*cs*-S-M) shoots and roots and associated GO terms. (Related to Fig. 4F, Supplementary Fig. S7E).

**Supplementary Data Set 16.** Gene lists and GO analyses of DEGs commonly dysregulated between photoautotrophic *cs* and heterotrophic WT shoots (*cs*-S-P and S-H) or roots (*cs*-R-P and R-H). (Related to Fig. 4I, Supplementary Fig. S8).

**Supplementary Data Set 17.** Metabolite profiles of WT and *clf swn* (*cs*) mutant seedling shoots. (Related to Fig. 5C, Supplementary Fig. S9, D—F).

**Supplementary Data Set 18.** Metabolite profiles of *A. thaliana* WT seed development stages. (Related to Fig. 5D, Supplementary Fig. S10A).

**Supplementary Data Set 19.** Concatenated numerical data set for the comparative correlation analysis (Supplementary Data Set 21) of WT and cs mutant profiles with a time series of *A. thaliana* seed maturation and germination. (Related to Fig. 5D).

**Supplementary Data Set 20.** Clustering of metabolites based on min-max-scaled abundance in *A. thaliana* seed maturation and germination. (Related to Supplementary Fig. S10, B—E).

**Supplementary Data Set 21.** Spearman correlation between the primary metabolomes of photoautotrophic (P) and mixotrophic (M) WT and *cs* shoot samples and samples representing *A. thaliana* seed maturation and germination. (Related to Fig. 5D).

**Supplementary Data Set 22.** List of genes upregulated in mixotrophic *cs* shoots (cs-S-M) and repressed by H3K27me3 in 3-DAG WT shoots (S-P3). (Related to Fig. 6A).

**Supplementary Data Set 23.** Genes upregulated in photoautotrophic *cs* shoots (cs-S-P/S-P) and marked by H3K27me3 in 3- (S-P3) and/or 7-DAG (S-P) WT shoots. (Related to Fig. 6B).

**Supplementary Data Set 24.** Overlap between genes upregulated in photoautotrophic or mixotrophic *cs* shoots compared with the respective WT shoots (*cs-*S-P/S-P or *cs*-S-M/S-M) with genes upregulated in LEC1_Oxi—*p*ER8-LEC1 (Mu et al. 2008) and genes involved in reserve storage catabolic pathways. (Related to Supplementary Fig. S11A).

**Supplementary Data Set 25.** List of primers used in this study.

**Supplementary Data Set 26.** Source data used to plot charts and heat maps.

## Dive Curated Terms

The following phenotypic, genotypic, and functional terms are of significance to the work described in this paper:

REF6 Gramene: AT3G48430
REF6 Araport: AT3G48430

AZF1 Gramene: AT5G67450
AZF1 Araport: AT5G67450

STM Gramene: AT1G62360
STM Araport: AT1G62360

FLC Gramene: AT5G10140
FLC Araport: AT5G10140

LEC2 Gramene: AT1G28300
LEC2 Araport: AT1G28300

AGL15 Gramene: AT5G13790
AGL15 Araport: AT5G13790

PCK1 Gramene: AT4G37870
PCK1 Araport: AT4G37870

ABI3 Gramene: AT3G24650
ABI3 Araport: AT3G24650

MAF4 Gramene: AT5G65070
MAF4 Araport: AT5G65070

MAF5 Gramene: AT5G65080
MAF5 Araport: AT5G65080

ELIP1 Gramene: AT3G22840
ELIP1 Araport: AT3G22840

AP2 Gramene: AT4G36920
AP2 Araport: AT4G36920

BLH2 Gramene: AT4G36870
BLH2 Araport: AT4G36870

ELIP2 Gramene: AT4G14690
ELIP2 Araport: AT4G14690

FIE Gramene: AT3G20740
FIE Araport: AT3G20740

AP1 Gramene: AT1G69120
AP1 Araport: AT1G69120

BLH4 Gramene: AT2G23760
BLH4 Araport: AT2G23760

KNAT4 Gramene: AT5G11060
KNAT4 Araport: AT5G11060

PP2A Gramene: AT1G13320
PP2A Araport: AT1G13320

SEP1 Gramene: AT5G15800
SEP1 Araport: AT5G15800

VAL1 Gramene: AT2G30470
VAL1 Araport: AT2G30470

h3k27me3 CHEBI: CHEBI:85045

ACT2 Gramene: AT3G18780
ACT2 Araport: AT3G18780

LHCA2 Gramene: AT3G61470
LHCA2 Araport: AT3G61470

JMJ32 Gramene: At3g45880
JMJ32 Araport: At3g45880

CDKA;1 Gramene: AT3G48750
CDKA;1 Araport: AT3G48750
