# Supplementary material for: Polycomb repressive complex 2 facilitates the transition from heterotrophy to photoautotrophy during seedling emergence
Source: Plant Cell. 2025 Jun 14;37(7):koaf148. doi: 10.1093/plcell/koaf148 (PMC12236341; doi:10.1093/plcell/koaf148)
Supplement: koaf148_Supplementary_Data [file koaf148_supplementary_data.zip › SupplementaryFiguresS1-S11.pdf]

Samo et al.: Polycomb Repressive Complex 2 facilitates the transition from heterotrophy to photoautotrophy during seedling emergence

# Supplementary Figures S1 – S11

Supplementary Figure S1

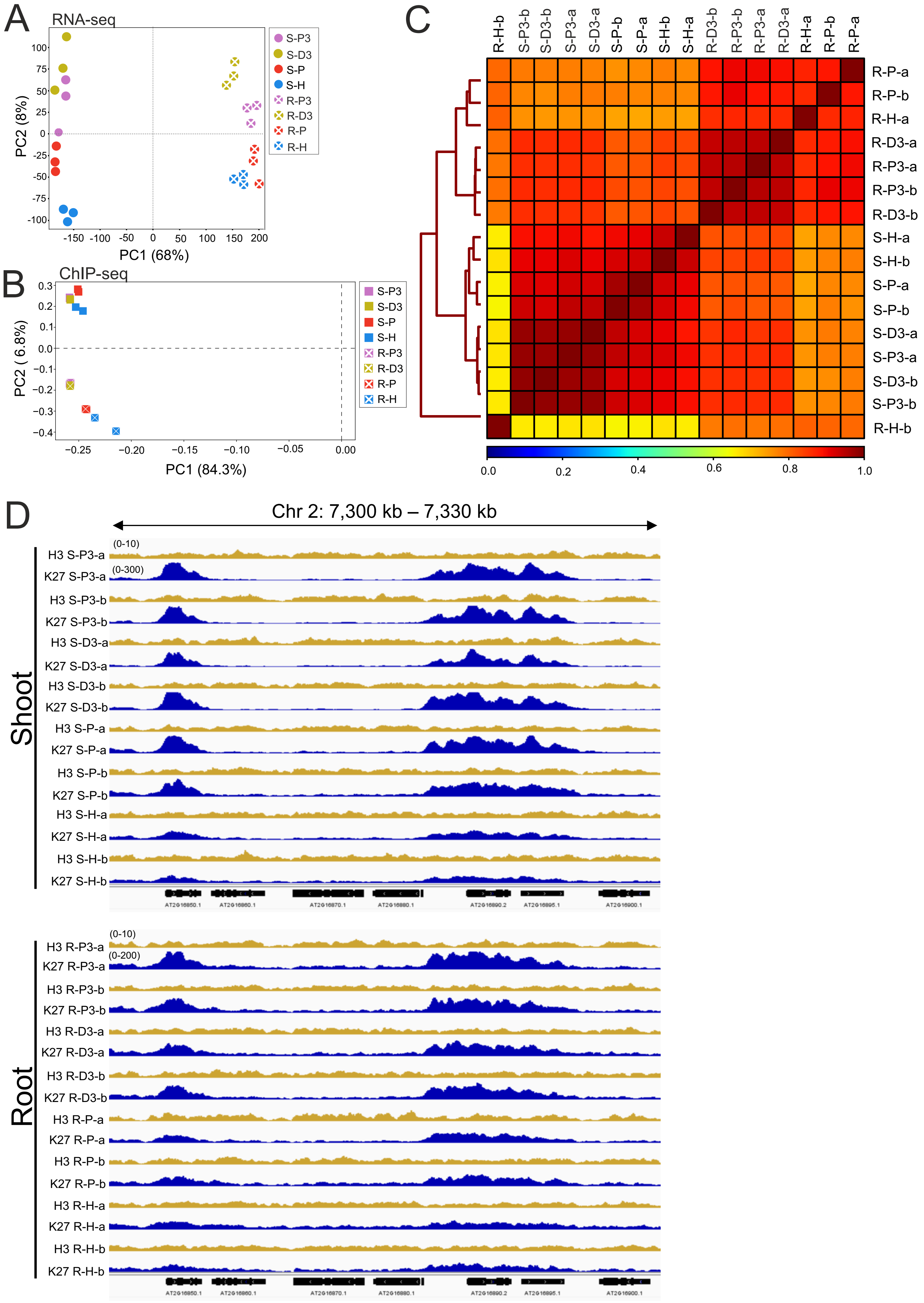

**Supplementary Figure S1. RNA-seq and ChIP-seq technical controls. (Supports Figure 1)**

**A)** RNA-seq principal component analysis (PCA). Reads Per Kilobase per Million mapped reads (RPKM) values of all genes were used; separate biological triplicates are shown. **B)** ChIP-seq principal component analysis (PCA) using average H3K27me3 BPM values over gene bodies of T70 genes (T70 in at least one of the samples); separate biological duplicates are shown. **C)** Spearman correlation of average H3K27me3 BPM values over gene bodies of T70 genes (T70 in at least one of the samples); separate biological duplicates (-a, -b) are shown. **D)** Genome browser display of a 30kb region in chromosome 2 showing the distribution of H3 (ochre) and H3K27me3 (blue, “K27”) in two replicates “a” and “b” of ChIP-seq of shoot and root samples. The Y-axis scale of all compared H3 and H3K27me3 (“K27”) tracks is identical and corresponds to the range indicated in the top tracks for shoot or root samples.

Supplementary Figure S2

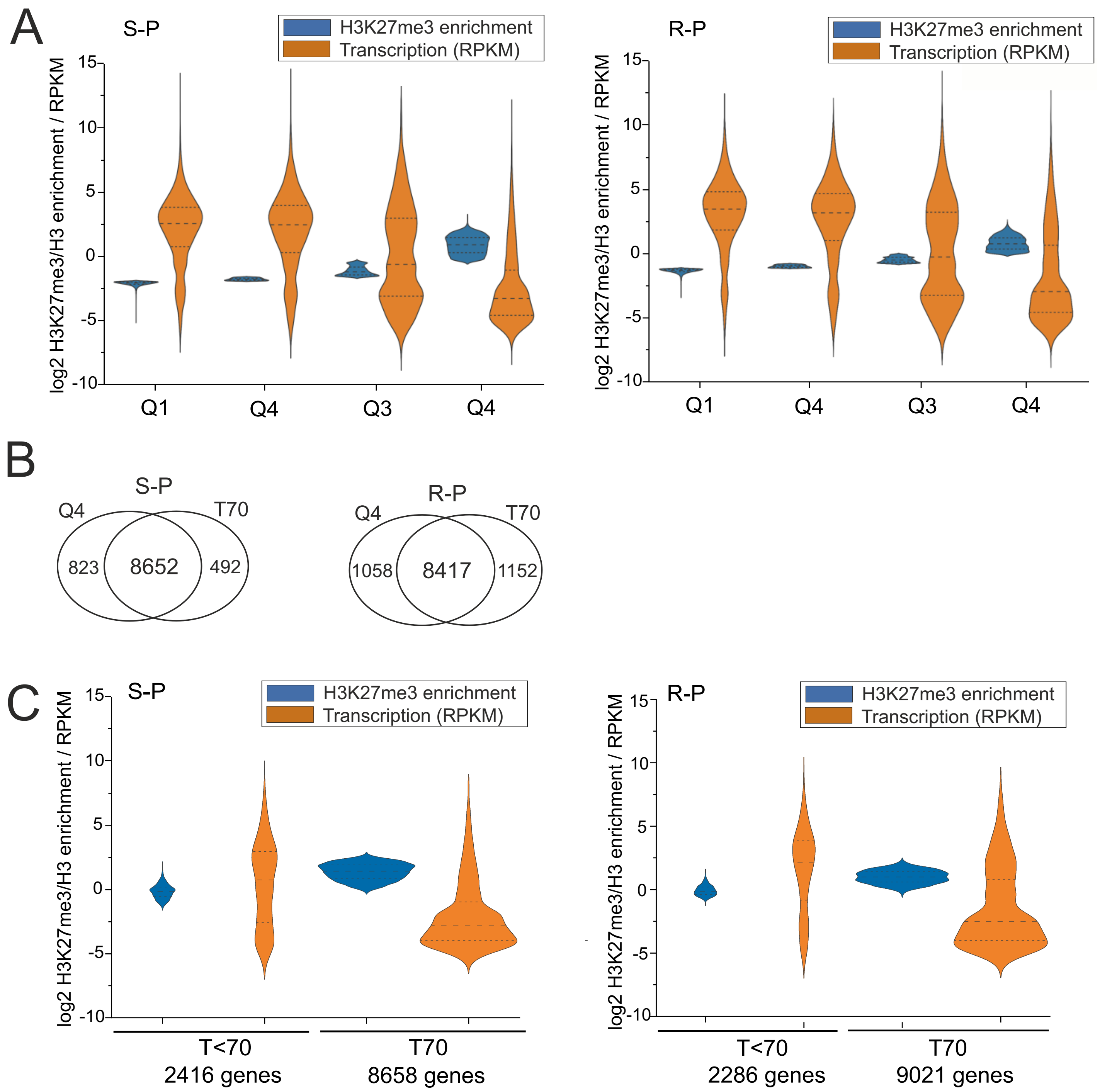

**Supplementary Figure S2. ChIP-seq technical controls: a high level of H3K27me3 enrichment is associated with low transcription. (Supports Figure 1)**

**A)** Association between H3K27me3/H3 enrichment and gene transcription (expressed as Reads Per Kilobase per Million mapped reads - RPKM). All Araport11-annotated genes (37392) were separated into quartiles (Q1 – Q4; N = 9348) based on the level of H3K27me3/H3 enrichment. Dashed horizontal lines represent mean (middle), first and third quartile. **B)** Overlap between genes identified as most-highly enriched for H3K27me3 (Q4) and genes with ≥70% gene body overlapping with H3K27me3/H3-enriched region (T70). **C)** Association between H3K27me3/H3 enrichment and gene transcription (RPKM) in photoautotrophic 7-DAG shoot and root tissues. T<70: genes with >0%<70% gene body or T70: genes with ≥70% gene body overlapping with H3K27me3/H3-enriched region. 7-DAG photoautotrophic shoot (S-P); 7-DAG photoautotrophic root (R-P). Similar patterns were observed for all samples. Dashed horizontal lines indicate mean (middle), 25% and 75% quartile.

Supplementary Figure S3

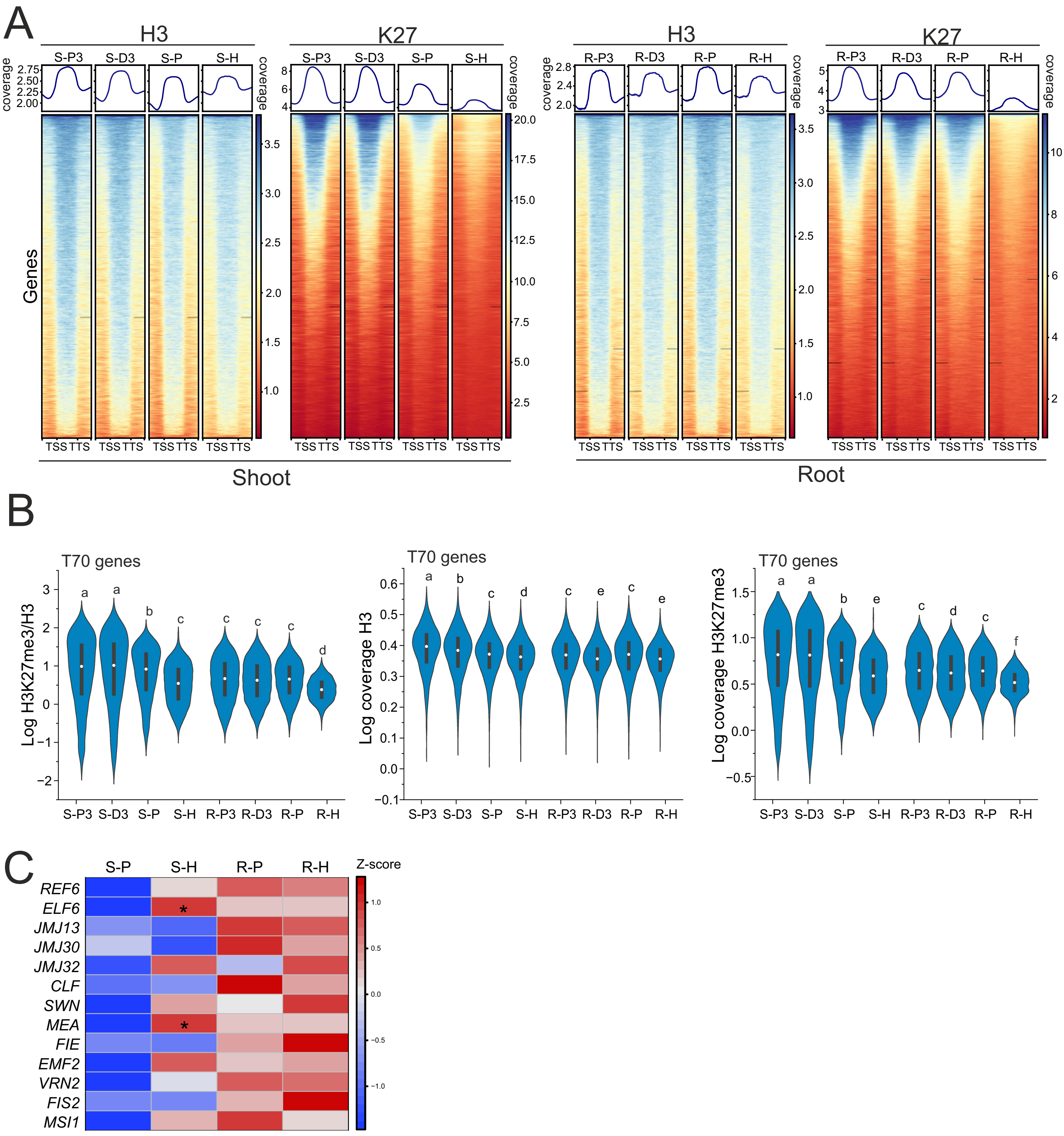

**Supplementary Figure S3. Coverage of H3 and H3K27me3 in H3K27me3 target genes identified in the shoot or root tissues. (Supports Figure 2)**

**A)** Coverage of H3 and H3K27me3 in gene bodies (-/+ 0.6 kb) in shoot and root samples for Araport 11–annotated genes. TSS – transcription start site; TTS – transcription termination site. **B)** Enrichment of H3K27me3/H3 or coverage of H3 and H3K27me3 in genes identified as H3K27me3-targets (T70) in at least one of the profiled samples. White dots represent mean; black box represents first and third quartiles. Letters above the violin distribution plots: significance levels, $p < 0.01$: Dunn´s multiple comparison test following Kruskal-Wallis ANOVA. N (T70 genes) = 8019 (S-P3); 7801 (S-D3); 8658 (S-P); 8152 (S-H); 8238 (R-P3); 8188 (R-D3); 9021 (R-P); 9156 (R-H. Sample labels in A) and B) correspond to Fig. 1B. **C)** Relative transcription of JMJ-domain H3K27me3 demethylase genes and PRC2 subunit genes in 7-DAG photoautotrophic (P) or heterotrophic (H) shoot (S) or root (R). Heatmap represents Z-score normalized mean RPKM values. Asterisks (*): significant difference between respective (P) and (H) samples; $p < 0.05$: DESeq2; N = 3 biological replicates.

# Supplementary Figure S4

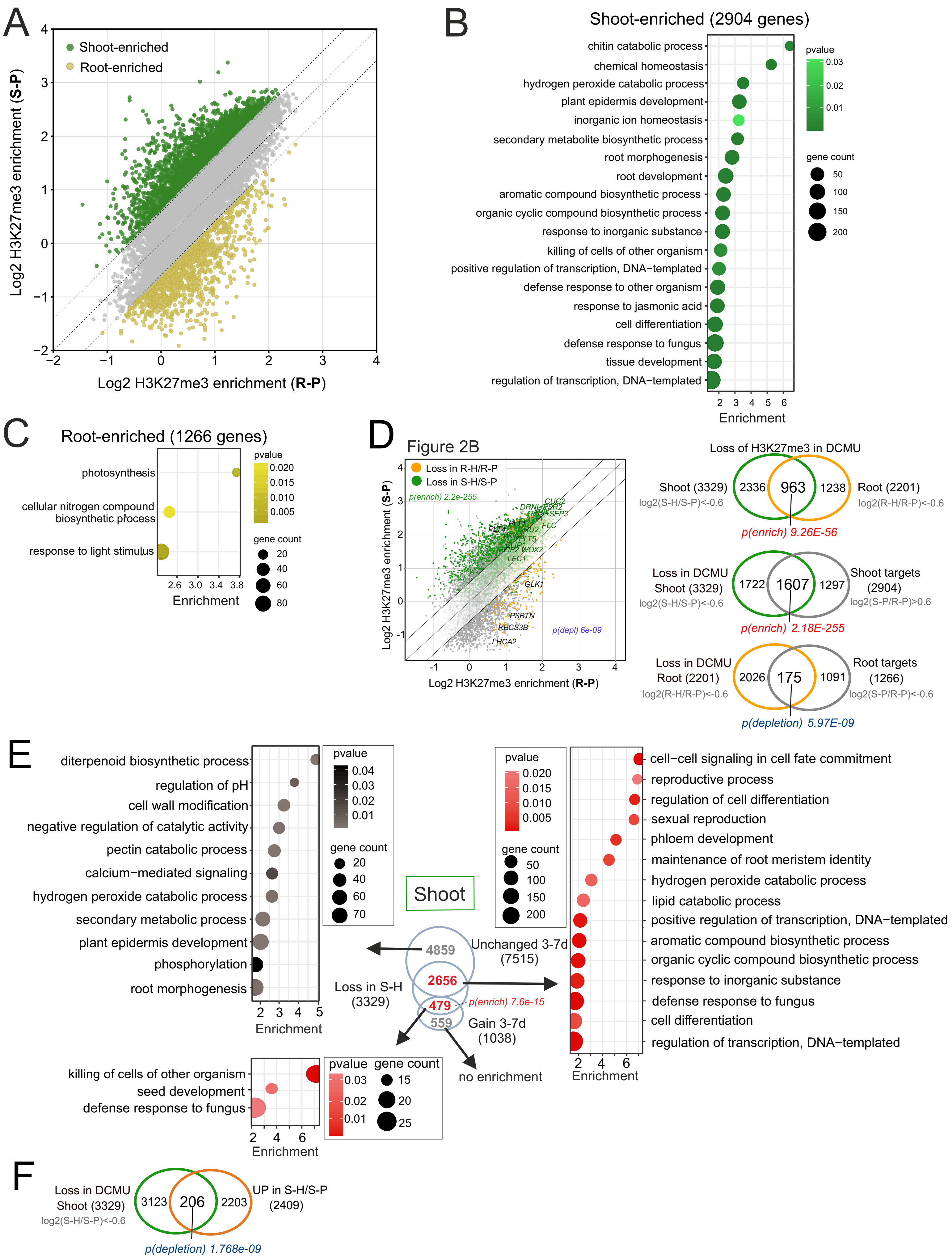

**Supplementary Figure S4. Shoot- and root-specific H3K27me3 targets and the impact of photosynthesis inhibition on H3K27me3 enrichment. (Supports Figure 2)**

**A)** H3K27me3/H3 enrichment of target genes in 7-day shoot (S-P) and root (R-P). Each dot represents a gene, all genes identified as H3K27me3-targets (T70) in either 7-DAG shoot or 7-DAG root are plotted. Green and ochre dots represent genes that are enriched for H3K27me3 in the shoot and root, respectively. **B)** GO analysis of genes enriched for H3K27me3 in the shoot only. **C)** GO analysis of genes enriched for H3K27me3 in the root only. BP categories are shown; GO display cutoff in B) and C): fold enrichment > 2; $p < 0.05$: hypergeometric test with Bonferroni correction. **D)** Extension to Fig. 2B. Left - Figure 2B reused for illustration: H3K27me3/H3 enrichment of target genes in 7-DAG shoot (S-P) and root (R-P). Each dot represents a gene; green and ochre dots represent genes that lose H3K27me3 in 7-DAG heterotrophic shoot (S-H/S-P) and root (R-H/R-P), resp. Venn diagrams on the right: p-values: hypergeometric test of significance of overlap (enrichment or depletion/impoverishment) between shoot (green) or root (ochre) genes and genes losing H3K27me3 in respective heterotrophic samples. **E)** GO enrichment of H3K27me3 targets with unchanged or gain of H3K27me3 between 3- and 7-DAG shoot (S-P3/S-P) that lose (red) or do not lose (grey) H3K27me3 in heterotrophic shoot (S-H). BP categories are shown; GO display cutoff: fold enrichment > 1.5; $p < 0.05$: hypergeometric test with Bonferroni correction. p(enrich): hypergeometric test of enrichment compared to expected. **F)** Overlap between genes that lost H3K27me3 and that were upregulated in heterotrophic shoot compared to photoautotrophic shoot (S-H/S-P). p(depletion): hypergeometric test of impoverishment compared to expected.

# Supplementary Figure S5

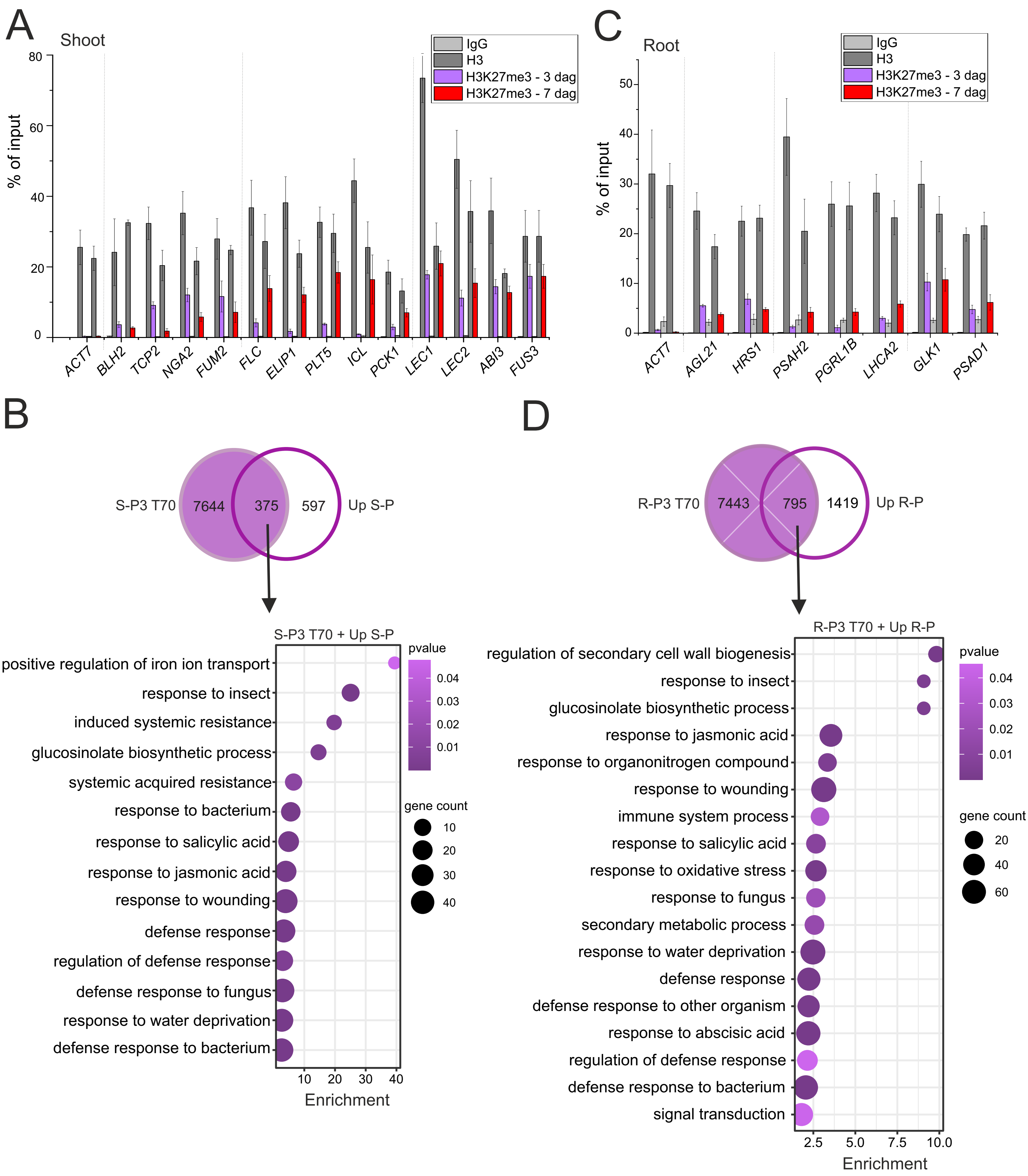

**Supplementary Figure S5. ChIP-qPCR confirmation of ChIP-seq and GO of genes that lose H3K27me3 and are activated between three and seven DAGDAG. (Supports Figure 3)**

**A)** Full version of Fig. 2C including ChIP controls: ChIP-qPCR analysis of representative genes with decreased (*BLH2, TCP2, NGA2* and *FUM2*), increased (*FLC, ELIP1, PLT5, ICL* and *PCK1*) or unchanged (*LEC1, LEC2, ABI3* and *FUS3*) levels of H3K27me3 from 3- to 7-DAG shoot. *ACT7* serves as negative control locus with no H3K27me3 enrichment. Bars: mean ±SD; N = 3 technical replicates. **B)** Gene ontology (GO) enrichment of genes that lose H3K27me3 and are transcriptionally upregulated between 3- and 7-DAG shoot. S-P3: 3-DAG shoot; S-P: 7-DAG shoot; T70: H3K27me3 target genes. BP categories are shown; GO display cutoff: fold enrichment > 1.5; $p < 0.05$: hypergeometric test with Bonferroni correction. **C)** Full version of Fig. 2H with ChIP controls: ChIP-qPCR analysis of representative genes showed decreased (*AGL21* and *HRS1*), increased (*PSAH2* and *PGRL1B*), and unchanged (*LHCA2, GLK1* and *PSAD1*) levels of H3K27me3 from 3- to 7-DAG root. *ACT7* serves as negative control locus with no H3K27me3 enrichment. Bars: mean ±SD; N = 3 technical replicates. **D)** GO enrichment of genes that lose H3K27me3 and are transcriptionally upregulated between 3- and 7-DAG root. R-P3: 3-DAG root; R-P: 7-DAG root; T70: H3K27me3 target genes. BP categories are shown; GO display cutoff: fold enrichment > 1.5; $p < 0.05$: hypergeometric test with Bonferroni correction.

# Supplementary Figure S6

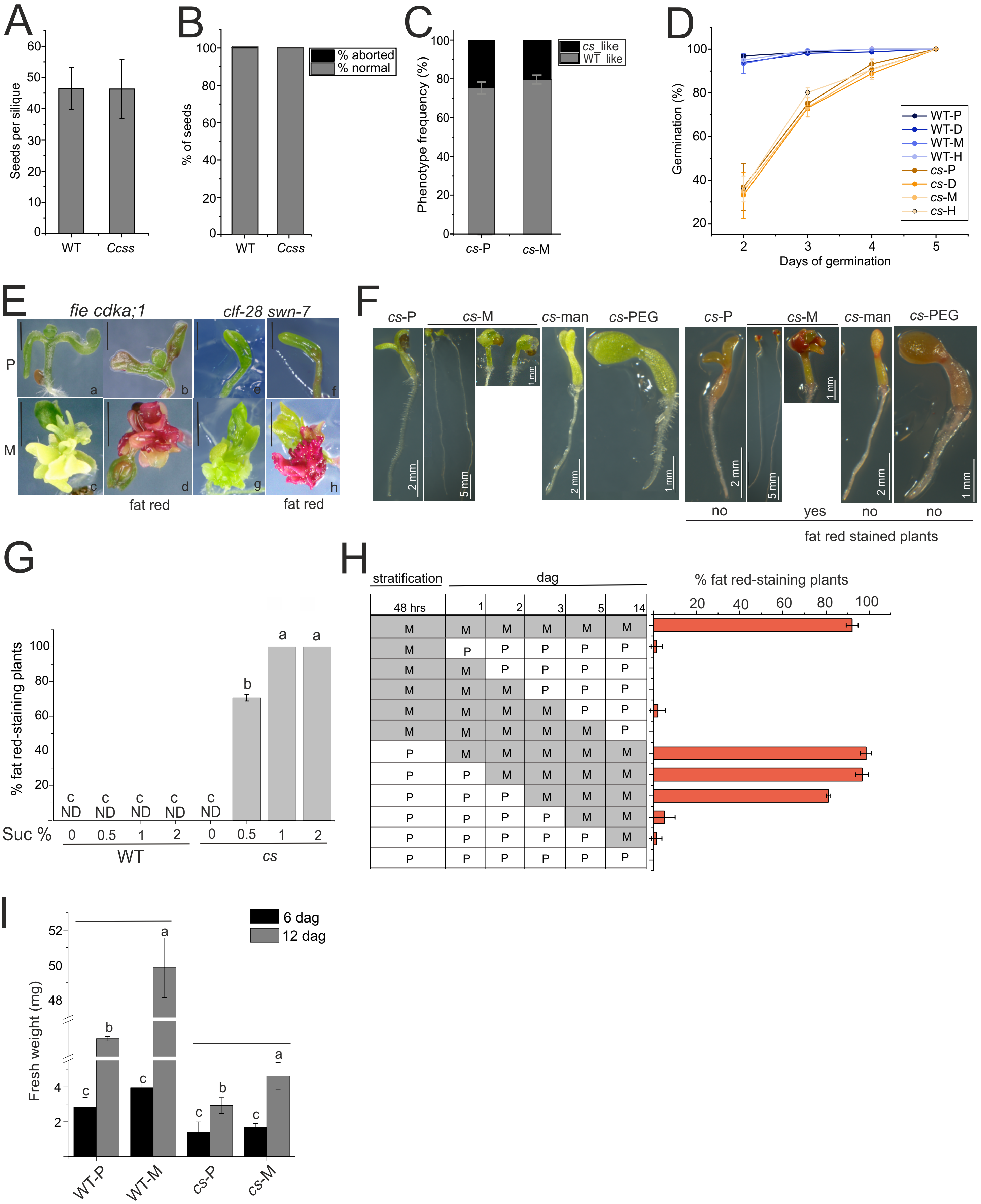

**Supplementary Figure S6. The absence of CLF and SWN does not reduce embryo or seed viability but is associated with delayed seed germination and sucrose-dependent triacylglycerol (TAG) accumulation. (Supports Figure 4)**

**A)** Seed set in WT and *CLF/clf swn/swn* (*Ccss*). Bars: mean ±SD; N = 58 WT and 80 *Ccss* siliques. **B)** Seed abortion rate in WT and *Ccss*. N = 4331 seeds (96 siliques, 17 individual plants) in WT and 6346 seeds (145 siliques, 15 individual plants) in *Ccss*. 0.23% and 0.34 % seeds aborted in WT and *Ccss*, resp. **C)** Penetrance of *clf swn* (*cs*) phenotype in 10-DAG progeny of *Ccss* plants cultivated in mixotrophic (*cs*-M) or photoautotrophic (*cs*-P) conditions. The frequency does not deviate from the expected 25% (p 0.912, 0.991 for M, P respectively; $\chi 2$ test). Bars: mean ±SD; N = 6 biological replicates (130 - 260 seeds/replicate/growth condition). **D)** Cumulative percentage of germination of WT and *cs* in photoautotrophic (WT-P, *cs*-P), mixotrophic (WT-M, *cs*-M), heterotrophic (WT-H, *cs*-H: +DCMU+suc) and +DCMU (WT-D, *cs*-D) conditions. Data points: mean ±SD; N = 2 biological replicates (seeds/replicate/time point: 133 – 208 in WT and 31 - 62 in *cs*). **E)** Phenotype of *fie cdka;1* (a - d) and *clf-28 swn-7* (e - h) seedlings grown in photoautotrophic (P) or mixotrophic (M) conditions. Fat red – plants stained by SudanRed7B to detect TAGs. Scale bar = 1 mm. N = 3 biological replicates (40- 50 *clf swn*). Penetrance of *fie* was approx. 2% in mixotrophic and not distinguishable in photoautotrophic conditions. **F)** Accumulation of TAGs in *clf swn* induced by sucrose (mixotrophy – M) but not by mock treatment (photoautotrophy – P), or osmotic controls including mannitol (man) or polyethylene glycol (PEG) in the growth medium. 30 mM sugar was used. **G)** Penetrance of TAG-accumulating phenotype in populations of WT and *cs* plants grown in photoautotrophic (sucrose 0 %) and mixotrophic (sucrose 0.5%, 1% and 2%) growth conditions. Bars: mean ± SD; N = 3 biological replicates (25 - 59 *cs* or 200 WT 21-DAG seedlings/replicate). Letters above bars: statistical significance at $p < 0.05$; two-way ANOVA with Bonferroni post hoc test. ND - not detected. **H)** Sucrose-induced TAG accumulation in *cs* is conditioned by the presence of sucrose within the first 2-3 days after seed germination induction. Timescale in the table indicates timepoints at which germinating seeds were transferred between cultivation plates with (M - mixotrophic) or without (P - photoautotrophic) 1% sucrose. Bars: mean ± SD; N = 3 biological replicates (21-38 14-DAG *cs* plants/replicate). **I)** Biomass of WT and *cs* seedlings cultivated in photoautotrophic (WT-P, *cs*-P) and mixotrophic (WT-M, *cs*-M) growth conditions for 6 and 12 days. Bars: mean ± SD; N = 4 biological replicates (5 seedlings/replicate were pooled). Letters above bars: statistical significance at $p < 0.05$; two-way ANOVA with Bonferroni post hoc test.

Supplementary Figure S7

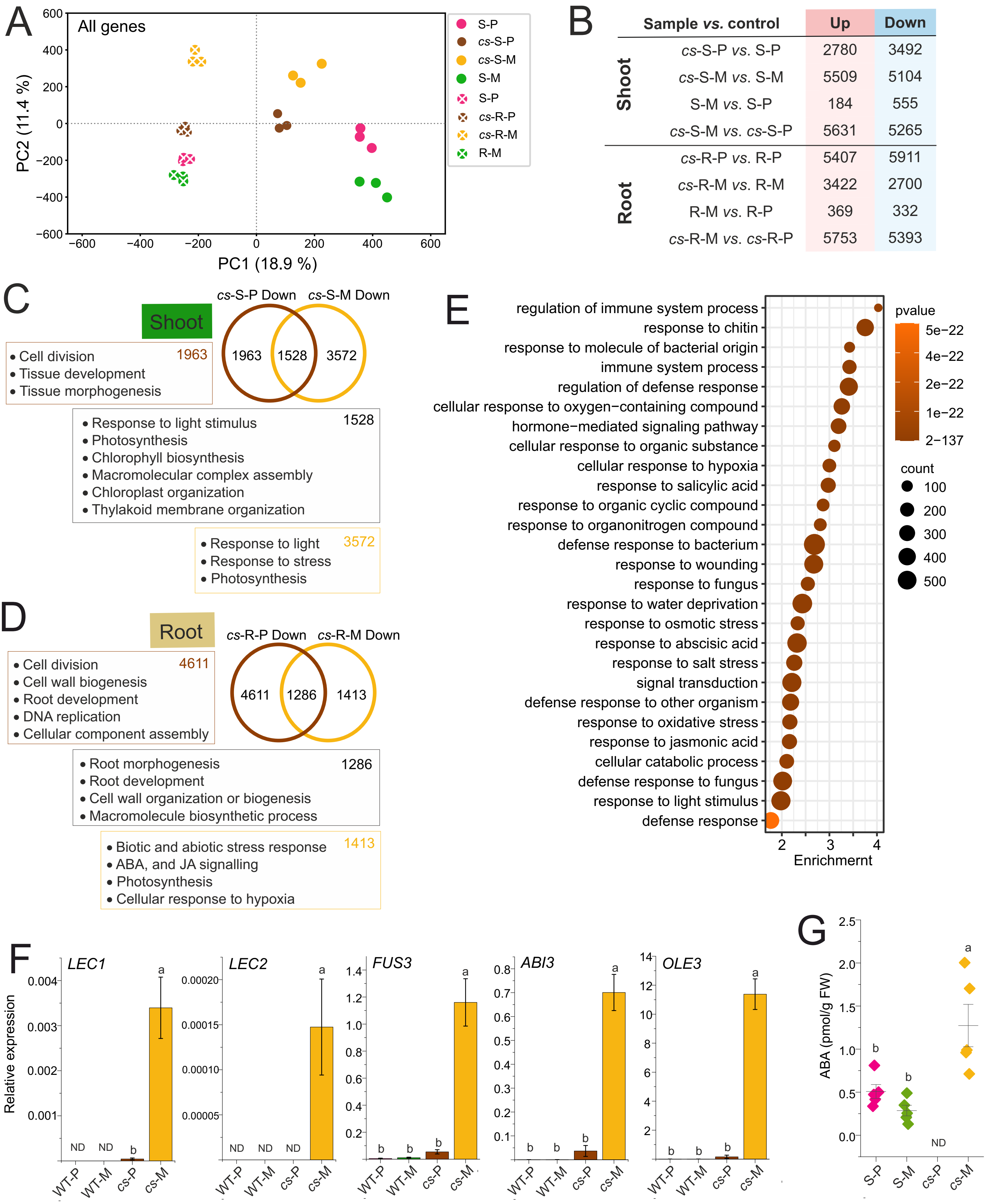

**Supplementary Figure S7. Transcriptome analysis of *clf swn* cultivated under photoautotrophic or mixotrophic conditions and ABA concentration in the samples. (Supports Figure 4)**

**A)** RNA-seq principal component analysis (PCA): Reads Per Kilobase per Million mapped reads (RPKM) of all ARAPORT11 genes were used. Samples: wild-type (WT) and *clf swn* (*cs*); photoautotrophic (P) and mixotrophic (M); shoot (S) and root (R) tissues. **B)** Numbers of differentially expressed genes (DEGs) in the analysed sample comparisons. Cutoff: absolute log2FC > 0.6, FDR < 0.05 (commonly identified in EdgeR and DESeq2). **C)** Schematic representation of genes and enriched GO biological processes downregulated in *cs* photoautotrophic (*cs*-S-P) and mixotrophic (*cs*-S-M) shoot compared to respective WT shoot (S-P and S-M) samples. GO summary threshold: $p < 0.05$: hypergeometric test with Bonferroni correction. **D)** Schematic representation of genes and enriched GO biological processes downregulated in *cs* photoautotrophic (*cs*-R-P) and mixotrophic (*cs*-R-M) root compared to respective WT root (R-P and R-M) samples. GO summary threshold: $p < 0.05$: hypergeometric test with Bonferroni correction. **E)** Biological processes enriched among genes upregulated in photoautotrophic (*cs*-S-P) compared to mixotrophic (*cs*-S-M) *cs* shoot. GO display cutoff: p < 1e-21: hypergeometric test with Bonferroni correction. **F)** Transcript abundances of *LEC1, LEC2, FUS3, ABI3* and *OLE3* determined by RT-qPCR wild type (WT) and *clf swn* (*cs*) under photoautotrophic (P) and mixotrophic (M) growth conditions. Bars: mean ± SD; N = 3 biological replicates. Letters above bars: statistical significance at $p < 0.05$; two-way ANOVA with Bonferroni post hoc test. **G)** Concentration of ABA in WT and *cs* shoot at 10 days. Bars: mean ± SD of 5 biological replicates. Letters above bars: statistical significance at $p < 0.05$; two-way ANOVA with Bonferroni post hoc test. ND - not detected.

Supplementary Figure S8

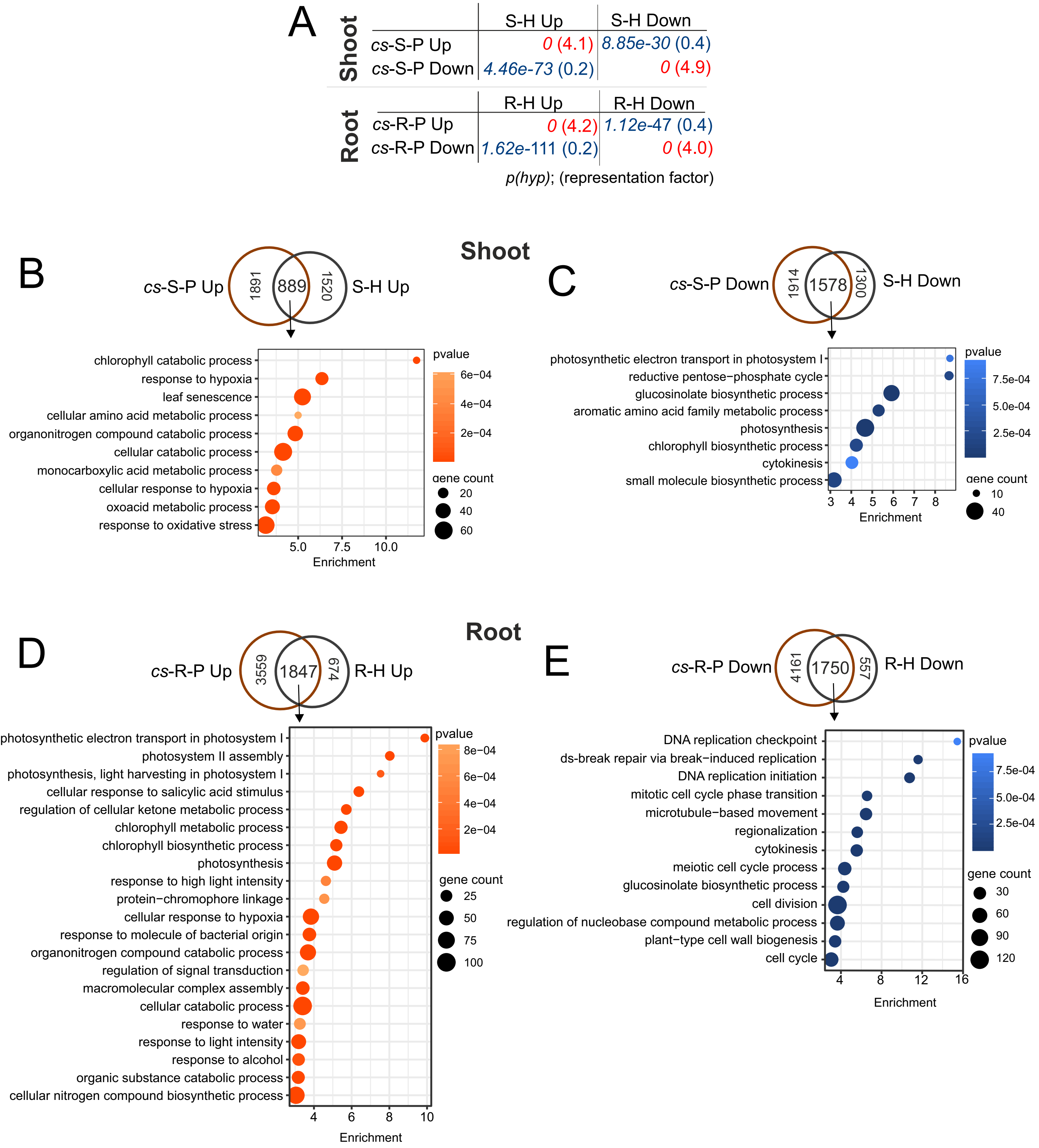

**Supplementary Figure S8. GO enrichment of the biological processes commonly up- or down-regulated in photoautotrophic *cs* and DCMU-grown WT. (Supports Figure 4H)**

**A)** P-values of hypergeometric tests for overlaps and (representation factor) of dysregulated gene sets in photoautotrophic *cs* (*cs*-S/R-P) and heterotrophic WT (S/R-H) shoot (S) and root (R) tissues. Representation factor indicates enrichment (red font) or impoverishment (blue font) compared to expected. **B-E)** Biological processes enriched among: **B)** genes commonly upregulated in photoautotrophic *cs* shoot (*cs*-S-P) and heterotrophic, DCMU-grown WT shoots (S-H); **C)** genes commonly downregulated in photoautotrophic *cs* shoot (*cs*-S-P) and heterotrophic, DCMU-grown WT shoots (S-H); **D)** genes commonly upregulated in photoautotrophic *cs* root (*cs*-R-P) and heterotrophic, DCMU-grown WT roots (R-H); **E)** genes commonly downregulated in photoautotrophic *cs* root (cs-R-P) and heterotrophic, DCMU-grown WT roots (R-H). GO display cutoff: fold enrichment > 3; $p < 0.001$: hypergeometric test with Bonferroni correction.

Supplementary Figure S9

A

| Sample | Genes |
|---|---|
| Up *cs*-S-P | 2780 |
| Up *cs*-S-M | 5506 |
| DS | 3198 |
| 6H | 1514 |
| TR | 43 |
| RP | 21 |
| RH | 35 |
| GC | 156 |
| OC | 1417 |

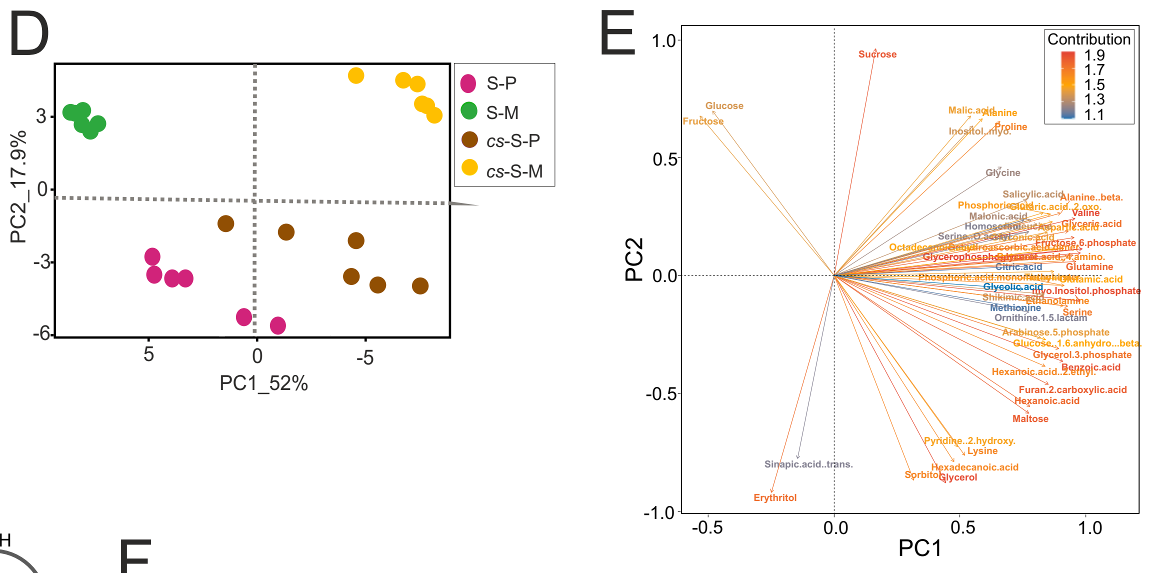


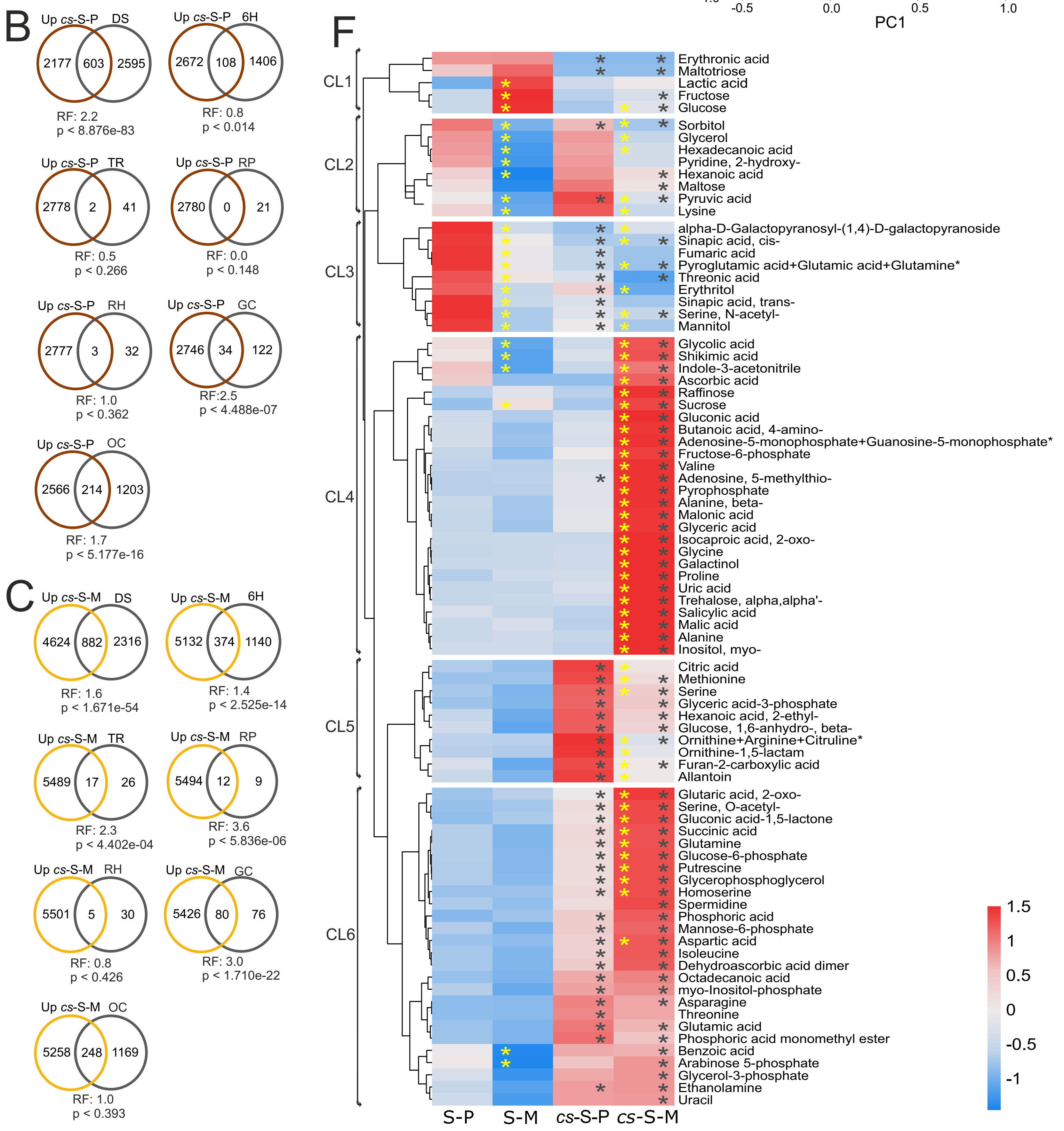

**Supplementary Figure S9. Comparison of transcriptome and primary metabolome data. (Supports Figure 5)**

**A)** Summary of compared genes. The numbers of genes reported by Silva et al. 2016 correspond to clusters of genes that have maximum expression at a given developmental stage. **B)** and **C)** Overlap analysis of numbers of differentially expressed genes in **B)** photoautotrophic (*cs*-S-P) and **C)** mixotrophic (*cs*-S-M) *cs* shoot and genes within clusters described by Silva et al. 2016. P-values of hypergeometric tests for gene set overlaps are shown; RF: representation factor indicates enrichment (RF > 1) or impoverishment (RF < 1) compared to random overlap. DS - dry seed, 6H - six hours of imbibition, TR - testa rupture, RP - radicle protrusion, RH - root hair emergence stage, GC - cotyledon greening stage, OC - open cotyledons stage. **D)** Principal Component Analysis (PCA) of metabolite profiles. Relative abundance data were normalized to sample fresh weight and internal standard, $\log_{10}$-transformed, mean-centred, and auto-scaled prior to PCA generation. Biplot of PC1 and PC2 with respective % of represented variance. **E)** Contribution plot of 55 metabolites contributing to PC1 and PC2. **F)** Heatmap of relative abundances of 85 metabolites in WT photoautotrophic (S-P) and mixotrophic (S-M) shoot and *cs* photoautotrophic (*cs*-S-P) and mixotrophic (*cs*-S-M) shoot samples (Means, n = 5 biological replicates, red-blue colour coded z-score normalized by row). Metabolites are sorted into 6 clusters by hierarchical cluster analysis to the left. Asterisks (*): statistical significance at $p < 0.05$ (adjusted) based on ANOVA and Tukey's HSD test. Black asterisks indicate significant difference among genotypes (*cs*-S-P vs. S-P and *cs*-S-M vs. S-M), yellow asterisks indicate significant differences induced by mixotrophy (S-M vs. S-P and *cs*-S-M vs. *cs*-S-P). Clusters (CL) 1-6 correspond to Fig. 5C.

# Supplementary Figure S10

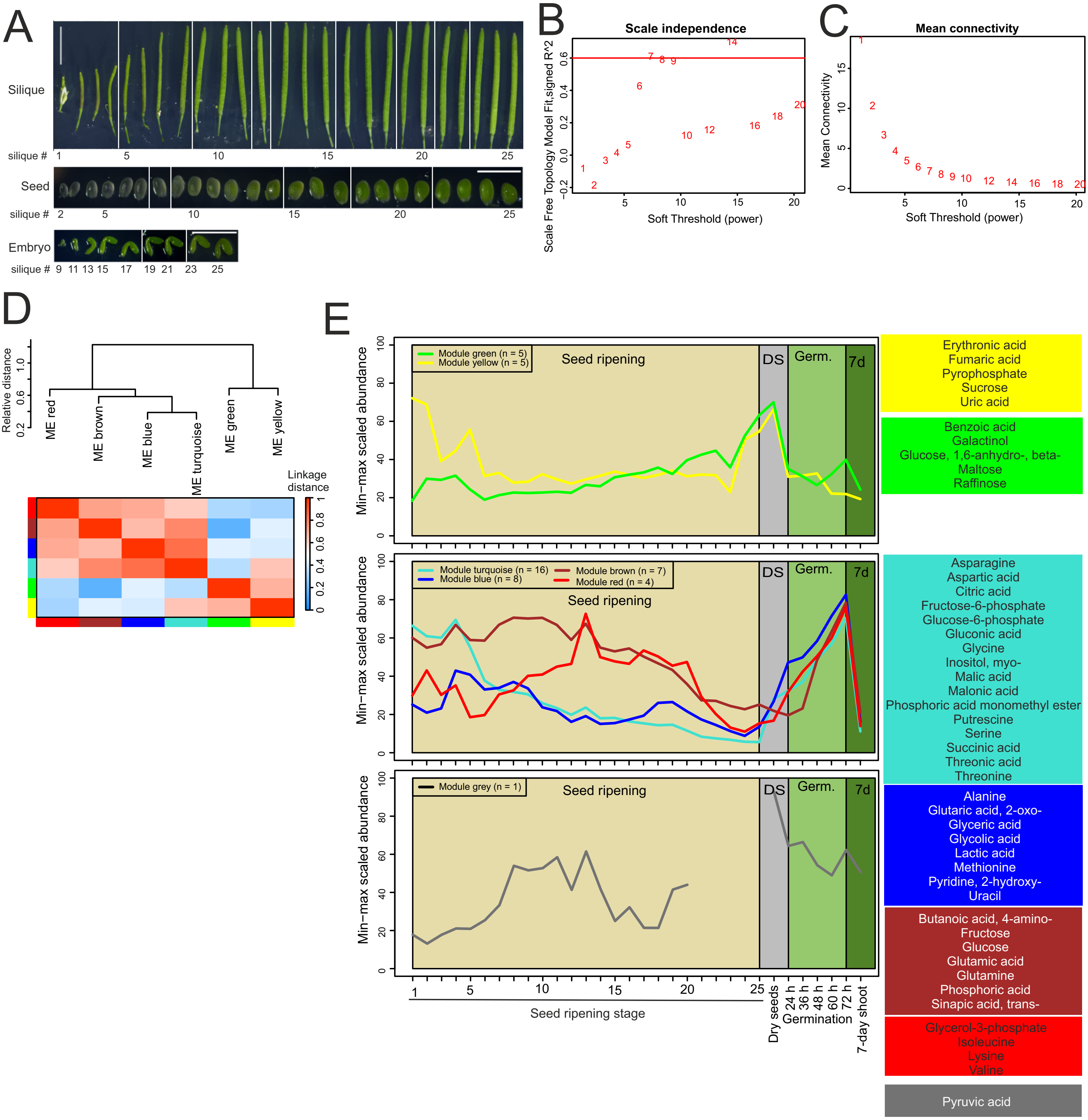

**Supplementary Figure S10. Weighted correlation network analysis of the primary metabolome profiles from 25 seed maturation stages, dry seeds, and five seed germination stages. (Supports Figure 5)**

**A)** 25 seed maturation stages of WT used for the profiling of the primary metabolome. Whole siliques were harvested at stages 1 – 6; seeds were harvested at stages 7 – 25. Seed maturation stages correspond to embryo development stages in the harvested material as shown by the bottom panel. Scale bar = 5 mm (applicable to the whole panel depicting siliques); 1mm (applicable to the whole panel depicting seeds and embryos). **B - E)** Weighted correlation network analysis (WGCNA) of 53 metabolites identified in samples of WT seed maturation stages. These metabolites were robustly present in all samples analyzed by this study. WGCNA was performed as follows: **B)** and **C)** Optimization of the soft-thresholding power β parameter for the weighted metabolites correlation network analysis (Langfelder and Horvath 2008). Weighted networks rely on metabolite-adjacencies, which correspond to the absolute correlation exponents of the soft-thresholding power β. The value β = 7 was chosen to obtain a network with Scale-Free Topology properties (Scale Free Topology Model Fit R2 ≥ 0.6) **(B)** and high connectivity **(C)**. **D)** Adjacency values were used to compute the Topological Overlap Matrix (TOM). Metabolites were clustered into modules (ME) based on the (1-TOM) distance matrix, by hierarchical clustering with average linkage distance comparison between clusters. The number of modules was determined by Dynamic Hybrid tree cut (dendrogram cut height for module merging = 0.25). **E)** Metamodules were identified based on the results of hierarchical clustering of the eigenmetabolites representing each ME, considering the (1-TOM) distance.

# Supplementary Figure S11

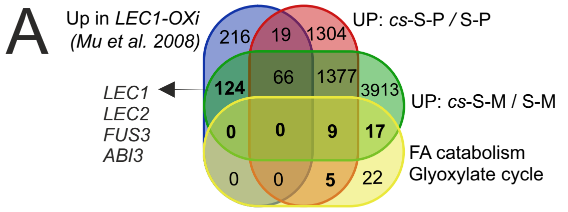


B

| Gene group | Description |
|---|---|
| 9036 genes | H3K27me3 target in 3 and/or 7-dag shoots (S-P3_T70 AND/OR S-P_T70) |
| 378 genes | H3K27me3 target only in 3-dag shoots (S-P3_T70 NOT S-P_T70) |
| 7641 genes | H3K27me3 target in 3 and 7-dag shoots (S-P3_T70 AND S-P_T70) |
| 1017 genes | H3K27me3 target only in 7-dag shoots (S-P_T70 NOT S-P3_T70) |
| 564 genes | **Fig. 6A**: H3K27me3 target in 3-dag shoot (S-P3_T70) and upregulated in cs-S-M, NOT upregulated in cs-S-P |
| 805 genes | **Fig. 6B**: genes equally enriched for H3K27me3 in 3 and 7-dag shoots (log2FC S-P3_T70/S-P_T70 > -0.6 < 0.6) AND upregulated in cs-S-P/S-P |
| 126 genes | **Fig. 6B**: genes enriched in H3K27me3 in 7-day shoot over 3/day shoot (log2FC S-P_T70/S-P3_T70 > 0.6) AND upregulated in cs-S-P/S-P |
| 8033 genes | H3K27me3 target in 3 and/or 7-dag shoots (S-P3_T70 AND/OR S-P_T70), NOT upregulated in cs-S-P/S-P |

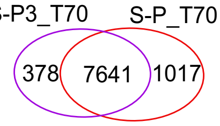


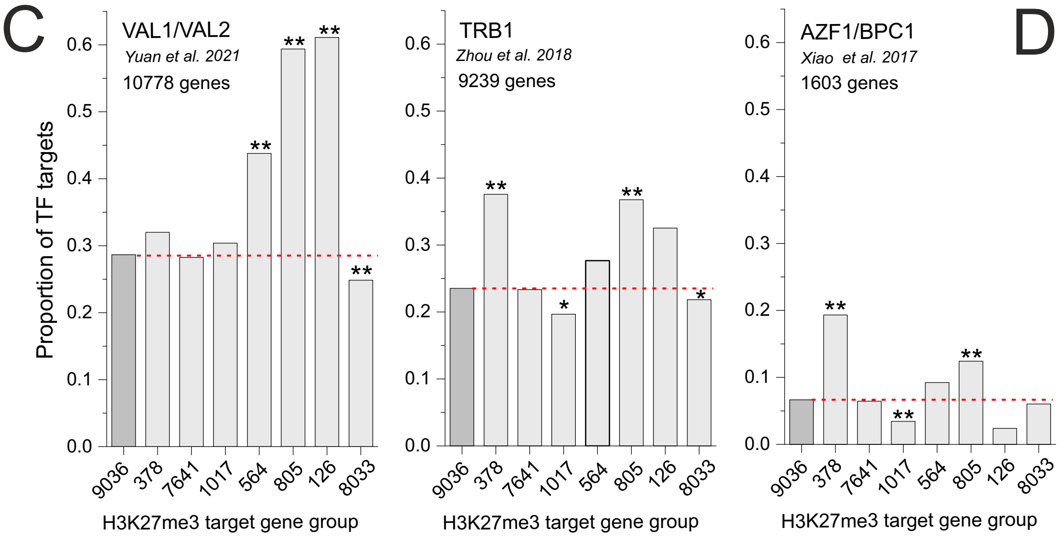


D

| Gene group | Motif | | p-value |
|---|---|---|---|
| 9036 genes | RY | CATGCA | 4.83e-37 |
| 378 genes | REF6 | CTCTGTT | 8.11e-03 |
| 7641 genes | RY | CATGCA | 9.24e-13 |
| | Telobox | AAACCCTAA | 1.63e-03 |
| 1017 genes | none | | > 0.05 |
| 564 genes | none | | > 0.05 |
| 805 genes | RY | CATGCA | 1.71e-05 |
| 126 genes | RY | CATGCA | 3.84e-02 |
| 8033 genes | RY | CATGCA | 3.75e-04 |
| | Telobox | AAACCCTAA | 3.14e-02 |

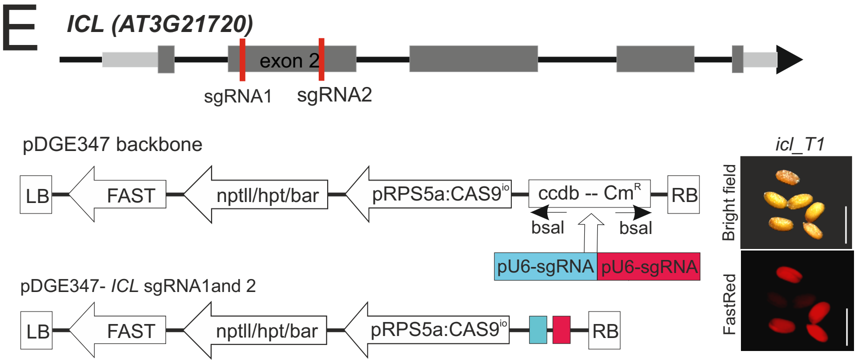


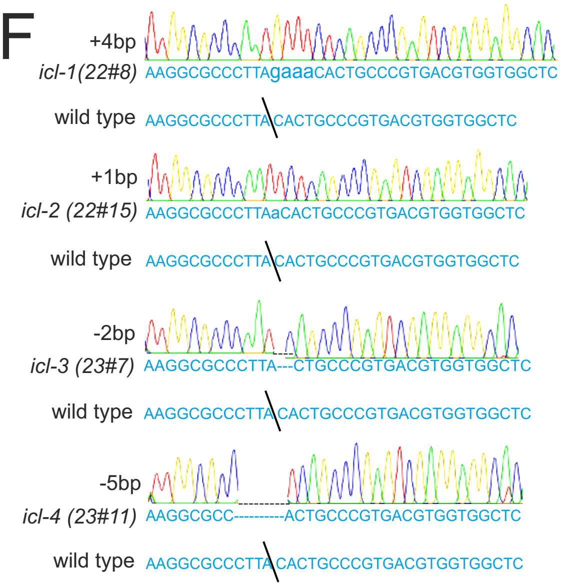


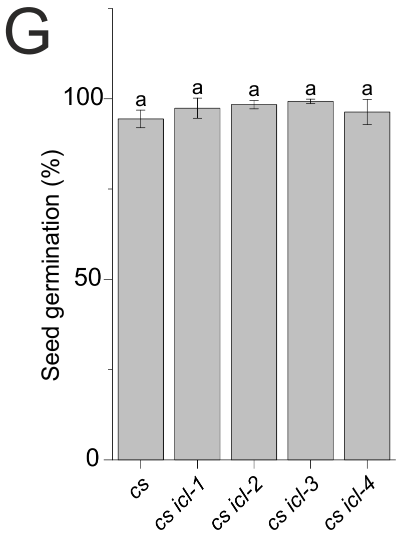


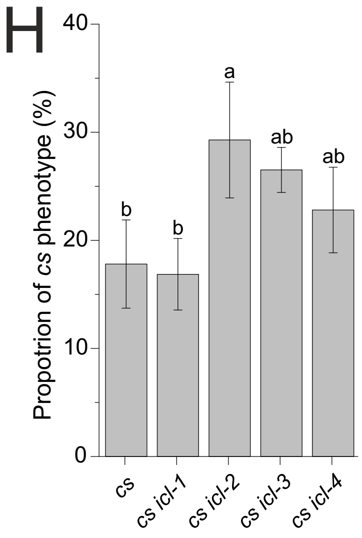


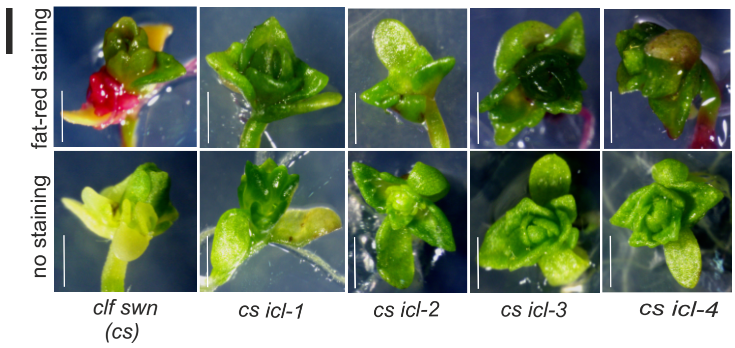

**Supplementary Figure S11. PRC2 recruitment modules involved in the seed-to-seedling transition and CRISPR/Cas9-mediated mutagenesis of *ICL* in *cs*. (Supports Figure 6)**

**A)** Overlap of genes upregulated in photoautotrophic (*cs*-S-P) or mixotrophic (*cs*-S-M) *cs* shoot compared to respective WT shoot controls, genes upregulated in *LEC1*-overexpressing plants (*LEC1-Oxi* - *pER8-LEC1*) compared to wild type (Mu et al. 2008) and genes involved in metabolic processes marking seed germination (the list of genes was downloaded from plant metabolic network (PMN: https://plantcyc.org/: fatty acid β-oxidation (BioCyc ID: PWY-5136), glyoxylate cycle (BioCyc ID: GLYOXYLATE-BYPASS), TCA cycle (BioCyc ID: PWY-5690), gluconeogenesis (BioCyc ID: GLUCONEO-PWY)). *LEC1-Oxi*-upregulated genes showed a significant overlap (hypergeometric test) with *cs*-M ($p < 6.27e-43$) and *cs*-P ($p < 3.95e-14$). *cs*-M ($p < 2.62e-05$) but also *cs*-P ($p < 0.00146$) showed an overlap with genes involved in seed germination-related metabolic pathways while none of these genes were upregulated in *LEC1-Oxi*. **B)** Groups and numbers of H3K27me3 target genes analysed in C and D. **C)** Proportion of transcription factor target genes among H3K27me3 targets. All H3K27me3 targets identified in 3-DAG and/or 7-DAG shoot (9036 genes) were used as background (dark grey column, indicated by red dashed line). Significant enrichment or depletion to background: *$p < 0.01$, **$p < 0.0001$; two-sided Fisher´s exact test. N = the number of genes analysed in each category corresponds to the name of the group as indicated on the x-axis; the total number of transcription factor target genes in the genome is as follows: VAL1/VAL2 – 10778 genes; TRB1 – 9239 genes; AZF1/BPC1 – 1603 genes. **D)** Polycomb response element (PRE)/REF6-motif enrichment among H3K27me3 targets. **E - I)** Generation and analysis of CRISPR/Cas9 *cs icl* lines. **E)** *ICL* (*AT3G21720*) locus and the positions targeted by the two guide RNAs (sgRNAs). *CRISPR/Cas9* construct used (Stuttmann et al. 2021) to transform *CLF/clf swn/swn* (*Ccss)* plants. Images on the right were digitally extracted for comparison. Scale bar = 1 mm. **F)** Sanger sequencing chromatogram and sequence of *ICL* in the four independent *clf swn* (*cs*) *icl* lines used. The gRNA sequence is shown in blue colour, the PAM sequence is shown as 3 bps in red colour, and black lines indicate the Cas9 cut site. Numbers in bp with (-) or (+) indicate deletion or insertion, respectively. **G)** – **I)** Analyses of T3 Cas9-negative plants in mixotrophic (1% sucrose) conditions. **G)** Seed germination in the progeny of *Ccss* plants is not affected by mutation in *ICL*. **H)** Frequency of *cs* phenotype is not reduced by *ICL* mutation in the progeny of *Ccss icl* plants. G) + H) Bars: mean ± SD; N = 3 biological replicates (95-105 seeds/replicate). Letters above bars: statistical significance at $p < 0.05$; one-way ANOVA with Bonferroni post hoc test. **I)** Representative images of *cs icl* plants analysed by fat-red staining – images complement statistics in Figure 6F. Scale bar = 1 mm (all images).
